# Effects of Transcranial Direct Current Stimulation Combined with Peripheral Electrical Stimulation on Upper Limb Function Recovery in Stroke: A Systematic Review and Meta-Analysis

**DOI:** 10.3390/brainsci16080791

**Published:** 2026-07-27

**Authors:** Ian Hoyin Cheng, Jibrin Sammani Usman, Shamay Sheung-Mei Ng

**Affiliations:** 1 Department of Rehabilitation Sciences, The Hong Kong Polytechnic University, Hong Kong, China; ian-hoyin.cheng@connect.polyu.hk (I.H.C.); jibrin.usman@polyu.edu.hk (J.S.U.); 2 Research Centre for Chinese Medicine Innovation, The Hong Kong Polytechnic University, Hong Kong, China

**Keywords:** transcranial direct current stimulation, tDCS, electrical stimulation, upper limb function, stroke rehabilitation

## Abstract

**Highlights:**

**What are the main findings?**

- Pairing tDCS and PES did not demonstrate clear significant effects on upper limb function after stroke when compared with tDCS alone and PES alone.
- Combining tDCS and PES with conventional rehabilitation demonstrated a potential ADL improvement after stroke when compared with conventional rehabilitation alone, which has a very low certainty of evidence and fragile.

**What are the implications of the main findings?**

- Current evidence, although of very low certainty, does not demonstrate a clear synergistic or additive effect of pairing tDCS and PES on upper limb motor function recovery after stroke.
- The potential benefit on ADL combining tDCS and PES with conventional rehabilitation must be interpreted with caution, and future studies are needed to further verify such potential impact.

**Abstract:**

Background/Objectives: Transcranial direct current stimulation (tDCS) and peripheral electrical stimulation (PES) have each demonstrated potential benefits for upper limb motor recovery in stroke patients. Their combined use has been hypothesized to produce synergistic/additive effects by engaging both central and peripheral neuroplasticity. This review aims to provide updated evidence on the effects of combined tDCS with PES on upper limb motor function and activity performance in stroke patients. Methods: Following PRISMA guidelines, six databases were searched. A systematic review and random-effects meta-analysis were completed. Methodological quality was assessed using the PEDro scale, risk of bias was assessed using the version 2 of the Cochrane risk-of-bias tool for randomized trials (RoB 2), and certainty of evidence was assessed using the GRADE approach. Results: Twelve RCTs involving 449 participants were included in this review. Adding motor-level PES to tDCS demonstrated a significant benefit on FMA-WH (MD = 1.53; 95% CI = 0.38 to 2.68; *p* = 0.009; low certainty; single study), while no significant effects on FMA-UE, MAS, ADL, and activity capacity were observed. Adding tDCS to motor-level PES yielded no significant benefits on FMA-UE and FMA-WH, while significant effects on handgrip strength and activity capacity were seen (with low certainty based on narrative reporting from individual studies). Adding tDCS to sensory-level PES showed no significant effects on FMA-UE, FMA-WH, MAS, muscle strength, and activity capacity. Adding tDCS and motor-level PES to standard therapy showed non-significant effect on FMA-UE, while significant benefits on FMA-WH (MD = 5.17; 95% CI = 4.09 to 6.25; *p* < 0.001), ADL (MD = 17.07; 95% CI = 15.54 to 18.60; *p* < 0.001), and MAL-AOU (with low certainty based on a single study) were observed. Adding tDCS and sensory-level PES to standard therapy demonstrated no significant overall effects on FMA-UE&LE (MD = 10.57; 95% CI = −32.29 to 53.43; *p* = 0.197, I^2^ = 43%), MAS (MD = −2.29; 95% CI = −13.09 to 8.51; *p* = 0.227; I^2^ = 57%), and ADL (MD = 12.16; 95% CI = −44.69 to 69.02; *p* = 0.224; I^2^ = 69%) (with low certainty based on multi-arms from a single study). Exploratory meta-analysis suggested a possible significant ADL improvement by combining tDCS and either motor/sensory PES with standard therapy (MD = 14.37; 95% CI = 1.83 to 26.91; *p* = 0.039; I^2^ = 71%)(with very low certainty). Conclusions: This review revealed that the current evidence, although of very low certainty, does not demonstrate a clear synergistic/additive effect of combined tDCS and PES on upper limb motor function recovery after stroke (which may be due to the substantial heterogeneity and various state-dependent responses across cohorts). Current evidence suggests a potential ADL improvement after stroke by pairing tDCS and PES with conventional rehabilitation; however, this was of very low certainty which requires future studies to further validate. Future studies should use factorial designs (comparing tDCS with PES, tDCS alone, PES alone, and sham or standard therapy control) to further verify the effect of pairing tDCS and PES on upper limb function after stroke.

## 1. Introduction

Stroke is the third leading cause of long-term disability worldwide, leading to persistent motor impairments that limit stroke survivors’ independence and quality of life [1]. Upper limb dysfunction is particularly prevalent after stroke, affecting up to 80% of stroke survivors in the acute phase and often persisting into the chronic phase [2]. Impairment in motor function hinders stroke patients’ ability to perform activities of daily living (ADL), contributing to loss of independence, reduced social participation, reduced quality of life, and increased caregiver burden.

Transcranial direct current stimulation (tDCS) is a non-invasive neuromodulation technique that delivers a low-intensity direct current to specific brain areas to modulate cortical excitability and neuronal activity [3]. Early applications of tDCS for stroke rehabilitation have demonstrated that anodal tDCS over the ipsilesional primary motor cortex (M1) could improve hand function in stroke patients [4], providing us with the foundation for subsequent clinical research on applying tDCS in stroke rehabilitation. Mechanistically, anodal stimulation of the ipsilesional motor cortex can facilitate excitatory plasticity, while cathodal stimulation of the contralesional hemisphere may reduce maladaptive interhemispheric inhibition [5,6]. Through modulation of cortical excitability in motor regions, previous studies have provided evidence of the therapeutic effect of tDCS alone on motor recovery [3,7,8,9,10,11]. However, despite initial promise, the clinical efficacy of tDCS alone in stroke rehabilitation remains controversial. Comprehensive evidence syntheses, such as the Cochrane review by Elsner et al. (2020), highlighted that while tDCS may improve ADL independence, its individual impact on upper limb function remains uncertain with variable trial outcomes [12]. Recent RCTs examining central stimulation in stroke rehabilitation have yielded similar heterogeneous and modest effects on motor recovery [13,14]. Thus, this ongoing uncertainty of tDCS neuromodulation in stroke rehabilitation implies the need to explore multimodal stimulations.

Peripheral electrical stimulation (PES), including modalities such as functional electrical stimulation (FES), neuromuscular electrical stimulation (NMES), and peripheral nerve stimulation (PNS), provides input to the sensorimotor circuits, reinforcing cortical reorganization through peripheral feedback [15]. Electrical stimulation (ES) is a widely applied clinical modality for sensory and motor stimulation. While sensory-level PES targets low-threshold cutaneous fibers to modulate pain or sensory processing without eliciting movement, motor-level modalities apply higher intensities to directly evoke muscle contractions for functional or rehabilitative training. Previous studies have reported that peripheral ES is a beneficial intervention for upper limb function recovery [16,17,18,19,20].

The combined application of tDCS and PES has been proposed to exert potential additive or synergistic effects by simultaneously engaging both central and peripheral neuroplasticity [21]. While some randomized controlled trials (RCTs) have suggested improvements in motor function recovery when pairing tDCS with PES, others have reported no significant benefits compared to single interventions or sham controls. In theory, PES generates ascending somatosensory impulses to the brain that stimulate descending corticospinal projections, modulate intracortical inhibitory circuits, and promote sensorimotor integration. The combined application of tDCS and PES is hypothesized to offer synergistic or additive therapeutic benefits by bridging the top-down central excitability modulation with bottom-up somatosensory feedback. This dual-stimulation approach is hypothesized to drive associative long-term potentiation (LTP)-like neuroplasticity, facilitate sensorimotor integration, reduce post-stroke spasticity, and enhance functional motor output beyond the independent impacts of either central or peripheral intervention [21]. However, whether this translates into true synergistic or additive clinical benefits remains inconclusive.

While the classical theory underlying combined tDCS and PES assumes an additive synergy between the top-down and bottom-up inputs, emerging neurophysiological evidence demonstrates that the nervous system’s response to external stimulation is fundamentally non-linear and depends on its baseline physiological state. Current neurophysiological frameworks suggest that the impact of concurrent central and peripheral stimulation is state-dependent and regulated by baseline cortical excitability and network-level neuroplasticity [22,23,24,25]. The combination of tDCS and PES may not generate uniform effects across all stroke populations, as therapeutic responsiveness may depend on factors like lesion site, corticospinal integrity, baseline excitability, post-stroke timing, and stimulation parameters, etc. For example, central neuromodulation protocols showed that localized stimulation generates widespread, network-level reconfigurations whose efficacy is heavily dependent on the baseline cortical excitability and age-dependent integrity of the neural system [26]. Therefore, apart from possible additive synergy, the state-dependent neuromodulation and network-level coordination between tDCS and PES are possible mechanisms that are worth exploring.

Although both tDCS and PES have been individually investigated in the rehabilitation of motor impairment, evidence regarding their combined application and effect remains limited and fragmented [27,28]. Therefore, a systematic review and meta-analysis is warranted to synthesize the current evidence, evaluate methodological quality, clarify whether combining tDCS and PES offers additive or synergistic effects to upper limb recovery after stroke, and explore the potential factors and mechanisms modulating the impact of the combined stimulation. Establishing the efficacy of this multimodal approach is crucial for guiding clinical practice and informing the design and interventional protocol of future RCTs.

To the best of our knowledge, and given the above-mentioned background, this is the first comprehensive systematic review and meta-analysis including quantitative analysis on the effects of tDCS combined with the full spectrum of PES modalities on upper limb function and activity recovery in stroke survivors.

## 2. Materials and Methods

**Figure 1:**
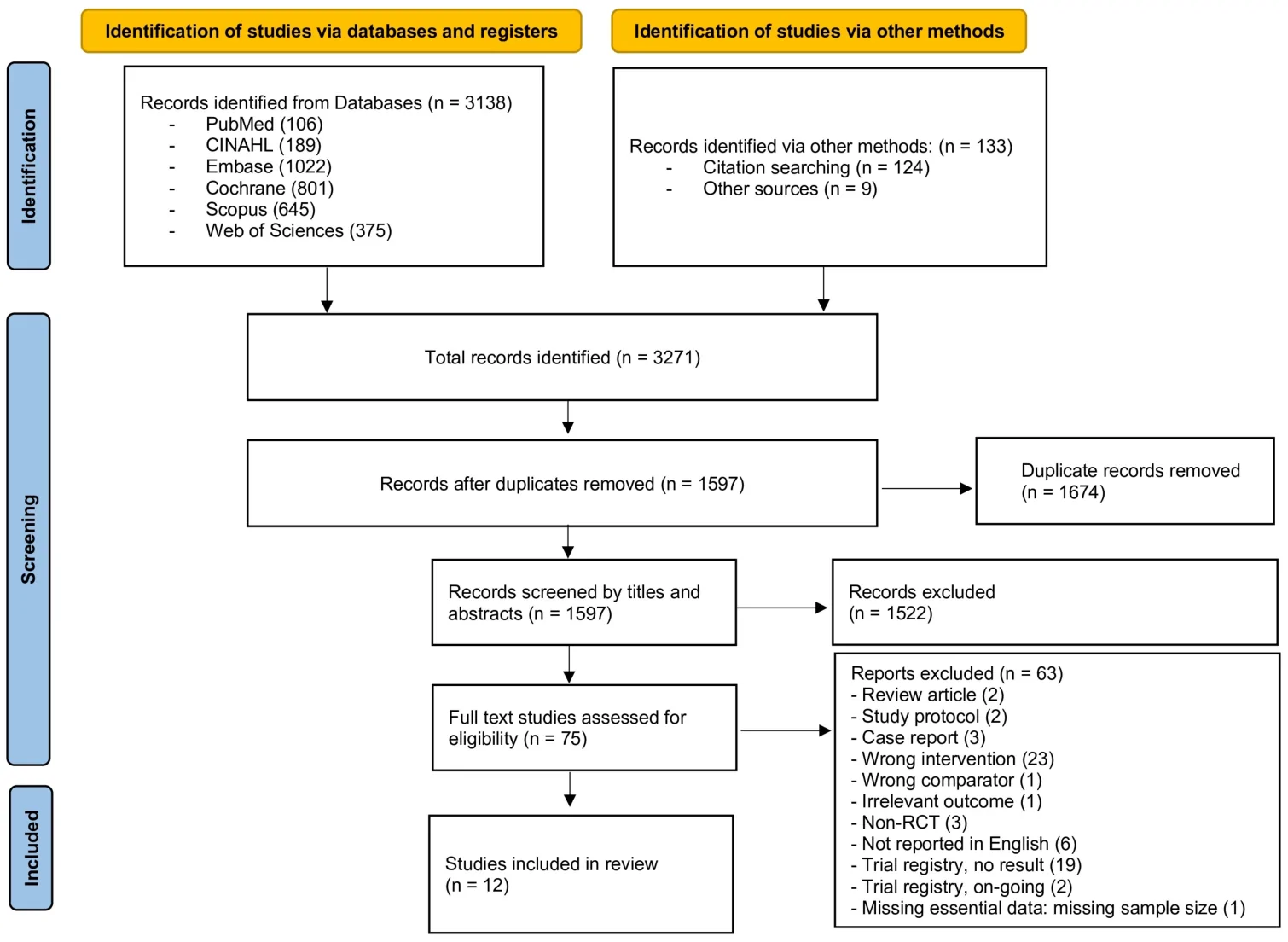
PRISMA flow diagram of the review process.

This systematic review and meta-analysis were conducted following the Preferred Reporting Items for Systematic reviews and Meta-Analyses (PRISMA) guidelines [29]. The completed PRISMA checklist is available in Supplementary Materials Table S1, and the PRISMA flowchart of the review process is presented in Figure 1. The review protocol was prospectively registered in PROSPERO (registration number: CRD420261309357) before the commencement of screening.

### 2.1. Eligibility Criteria

The PICOS framework was applied to establish and structure the eligibility criteria as follows.

#### 2.1.1. Population (P)

RCTs including patients with stroke, aged 18 years or above, with upper limb dysfunction after stroke were included.

#### 2.1.2. Intervention (I)

Interventions that combined active tDCS with active PES, where the PES must be applied to the paretic upper limb, with or without standard conventional rehabilitation therapy (CR), were included.

Motor-level and sensory-level PES modalities were treated as separate categories for primary findings interpretation. While a broad umbrella PES category encompassing both motor-level and sensory-level modalities was taken for exploratory analysis to explore the broad effects, as they all fundamentally generate ascending somatosensory afferent signals to modulate cortical excitability. 

#### 2.1.3. Comparison (C)

The following four comparison groups were included:

1. Active tDCS alone/active tDCS combined with sham PES with/without CR;
2. Active PES alone/sham tDCS combined with active PES with/without CR;
3. Combined sham tDCS and sham PES with/without CR;
4. Standard CR or other therapy alone.

#### 2.1.4. Outcome (O)

Outcomes related to upper limb function and activity levels were included. The outcome measures were organized into three domains of the ICF framework [30].

#### 2.1.5. Study Design (S)

RCTs with parallel or crossover designs were included. Only peer-reviewed studies published in English were included. There was no restriction on the publication period of studies to be included. 

#### 2.1.6. Excluded Studies

Reviews, meta-analyses, comments, case reports, conference abstracts, theses, and dissertations were excluded. Studies missing essential data for data synthesis were excluded. Non-English studies were excluded. 

### 2.2. Search Strategy

Six online databases, including PubMed, CINAHL Complete, Embase, Cochrane Library, Scopus, and Web of Science, were searched for relevant studies from inception to January 2026. The search strategy was compiled according to the PICOS strategy. Additional studies were identified by manual search of the references of relevant studies and reviews [18,27,28]. To identify completed but unpublished trial records, trial registries were searched via the Cochrane Library, which covered the Cochrane Central Register of Controlled Trials (CENTRAL) that includes clinical trial registries from ClinicalTrials.gov and the International Clinical Trials Registry Platform (WHO ICTRP). Studies, including gray literature and preprints, identified by a general literature search were screened. Identified studies were imported into a reference manager for removal of duplicates before title and abstract screening.

The search strategy was initially developed and conducted by one author (IHC). The complete search strategy, including search strings, keywords, Boolean operators, databases, and eligibility criteria, was independently checked, revised, and verified by the other two authors (JSU and SSMN) before the search. Details of the search strategy are shown in Supplementary Materials Table S2.

### 2.3. Selection of Studies

After removing duplicates, two authors (IHC and JSU) screened the titles and abstracts of the remaining studies according to the pre-specified eligibility criteria independently. After title and abstract screening, the full texts of studies were obtained for full-text screening against eligibility. Any disagreement regarding the eligibility of studies was resolved by discussion and consultation with the other author (SSMN). 

### 2.4. Data Extraction

Two authors (IHC and JSU) extracted the data independently by entering data into a standardized form on Microsoft Excel. Any disagreements regarding the extracted data were resolved by discussion and consultation with the other author (SSMN) to come to a consensus. If data were reported in figures and graphs only, the numerical values were pre-planned to be extracted by WebPlotDigitizer [31]. When data were missing, the study authors were contacted by email, whenever possible, to seek the missing data. Data of each included studies extracted include the author, publication year, design type, sample size, sex, age, stroke phase, duration since stroke, stroke type, affected side, intervention group(s), control group(s), outcomes, assessment time point, tDCS parameters (type of tDCS, device used, site, electrode size, intensity, duration of stimulation), PES parameters (type of PES, device used, site, electrode size, frequency, pulse width, duty cycle, intensity, duration), number of sessions and duration of stimulation, side effects, and results. 

### 2.5. Assessment of Methodological Quality

Methodological quality (MQ) of included studies was assessed by the PEDro scale [32]. The PEDro scale assesses the validity and quality of statistical reporting of studies by 11 items, which are rated dichotomously as 1 (Yes) or 0 (No), representing the presence or absence of evidence, respectively. By excluding the first item, which assesses the report of eligibility criteria, the PEDro scale ranges from 0 to 10. The total scores of 0–3, 4–5, 6–8, and 9–10 indicate poor, fair, good, and excellent MQ, respectively [33,34]. 

### 2.6. Assessment of Risk of Bias (ROB)

Risk of bias (ROB) in the included studies was assessed using version 2 of the Cochrane risk-of-bias tool for randomized trials (RoB 2) [35]. The RoB 2 is composed of five domains to assess the risk of bias of the study in bias arising from the randomization process, bias due to deviations from intended intervention, bias due to missing outcome data, bias in the measurement of outcomes, and bias in the selection of reported results. In addition, there is an additional domain assessing the bias arising from period and carryover effects for crossover-designed RCTs. By evaluating the study with a decision of high risk, some concern, or low risk of bias in each domain, an overall risk of bias (high risk, some concern, and low risk) in the included studies was obtained. Two independent authors (IHC and JSU) conducted the ROB evaluation. Any disagreements were resolved by consulting the third author (SSMN).

### 2.7. Data Analysis

Qualitative and quantitative syntheses were applied to analyze the extracted data.

For qualitative synthesis, the data were explained by the MQ, ROB, and characteristics of studies.

For quantitative syntheses, statistical analyses were performed using “meta” package in R (version 4.6.1) [36]. As the number of included studies in meta-analyses was very small (2 to 5 studies), restricted maximum likelihood (REML) with Hartung-Knapp (HK) adjustment was applied. Meta-analyses were conducted for outcomes that were reported by at least two studies with adequate data reported. Baseline characteristics between groups of each included study were reviewed to ensure that there were no baseline imbalances regarding demographic variables and baseline clinical outcomes. Post-intervention scores (mean and SD) were extracted and pooled for meta-analyses because change-score SDs were rarely reported in the included studies, and imputing correlation coefficients risked introducing mathematical estimation bias. Post-intervention score pooling is considered statistically appropriate under Cochrane guidelines given that baseline imbalances are absent and baseline comparability is confirmed [37]. The mean, SD of the outcomes at the post-intervention time point, and sample size of each group were pooled to conduct the meta-analyses. For outcomes assessed and reported by the same assessment tool, the mean difference (MD) and 95% confidence interval (CI) of a random-effects model were calculated. For outcomes assessing the same underlying construct but reported by different assessment scales, the standardized mean difference (SMD) and 95% CI of the random-effects model were calculated using Hedges’ g to correct for small-sample size bias.

When two comparisons from a single study were pooled in the same meta-analysis, the sample size of the shared group was split in half to avoid double-counting.

In cases where conducting a meta-analysis was not possible because of inadequate data to compute the meta-analysis, or due to the case that fewer than two studies reported the outcome, the effect size of the outcome was calculated using their mean and SD.

Only outcomes with complete data reported or data that could be retrieved by statistical methods were included in the quantitative analysis. Data reported with inadequate statistical details to compute the effect size were excluded from quantitative analysis and reported narratively.

The Cochrane GRADE approach was applied to assess the certainty of the evidence according to levels of risk of bias, inconsistency, indirectness, imprecision, and other considerations (such as publication bias). 

### 2.8. Assessment of Heterogeneity

I^2^ statistics were applied to assess the heterogeneity of the studies. The heterogeneity of studies was indicated by the value of I^2^. A value of 25%, 50%, and 75% presents a low, moderate, and high degree of heterogeneity, respectively [37].

To explore possible causes of heterogeneity, subgroup analyses were conducted. To preserve distinct clinical interpretations, subgroup analyses according to different variants of Fugl–Meyer Assessment (FMA) were conducted. To address clinical and parameter diversity across different PES modalities, subgroup analysis was established to separate and sensory-level and motor-level PES effects independently. Additionally, exploratory subgroup analyses evaluating the impact on FMA in different stroke phases, chronic and acute/subacute stroke, were conducted. While subgroup analysis on other outcomes were restricted by the limited number of pooled studies.

Other clinically critical moderators, such as tDCS montage, stimulation intensity, treatment sessions, and follow-up durations, were systematically screened during data extraction. Formal quantitative subgroup analyses based on these factors were not feasible due to the limited number of pooled studies per outcome. These potential sources of heterogeneity were discussed narratively. 

### 2.9. Sensitivity Analysis

Sensitivity analyses were conducted to evaluate the stability and robustness of the primary pooled estimates in the presence of high heterogeneity. Firstly, sequential “leave-one-out” analysis was applied to identify studies or individual comparisons that contributed disproportionately to heterogeneity or demonstrated an outlier effect. Secondly, analysis using the standard DerSimonian–Laird (DL) random-effects model via Review Manager 5.4 (Review Manager 5 (RevMan 5) [Computer program]. Version 5.4. Copenhagen: The Cochrane Collaboration, 2020) was applied to test the robustness of the primary pooled estimates.

## 3. Results

In the qualitative synthesis, data were synthesized narratively.

### 3.1. Identification and Selection of Eligible Studies

A total of 3271 studies were identified by searching databases (*n* = 3138) and other sources (*n* = 133), and 1674 duplicate studies were removed. By title and abstract screening of 1597 studies, 1522 studies were excluded according to the inclusion and exclusion criteria. In total, 75 full texts were assessed against the eligibility criteria, which led to an exclusion of 63 studies. Eventually, 12 studies were included in this review. Figure 1 shows the process of identification and selection of included studies according to the PRISMA guidelines.

### 3.2. Methodological Quality and Risk of Bias Analysis

#### 3.2.1. Methodological Quality Assessment by PEDro Scale

Details of the methodological quality (MQ) of the 12 included studies are presented in Table 1. Assessed via the PEDro scale, nine studies demonstrated good MQ and three achieved excellent MQ. All the included studies specified the eligibility criteria, conducted random allocation, involved groups similar at baseline in terms of important prognostic indicators, measured key outcomes from over 85% of the participants initially allocated to groups, reported statistical comparisons between groups for the key outcomes, and provided point measures and measures of variability for key outcomes. Allocation was concealed in six studies [38,39,40,41,42,43]. Regarding blinding, eight studies had subject blinding [38,39,40,42,44,45,46,47]. None of them had experimenter blinding. All studies except two [48,49] had adopted outcome assessor blinding. All studies except four [38,41,45,47] had available outcome measures from all subjects that received the treatment as allocated or had reported results of key outcomes by intention-to-treat analysis.

#### 3.2.2. Risk of Bias in the Included Studies

**Figure 2:**
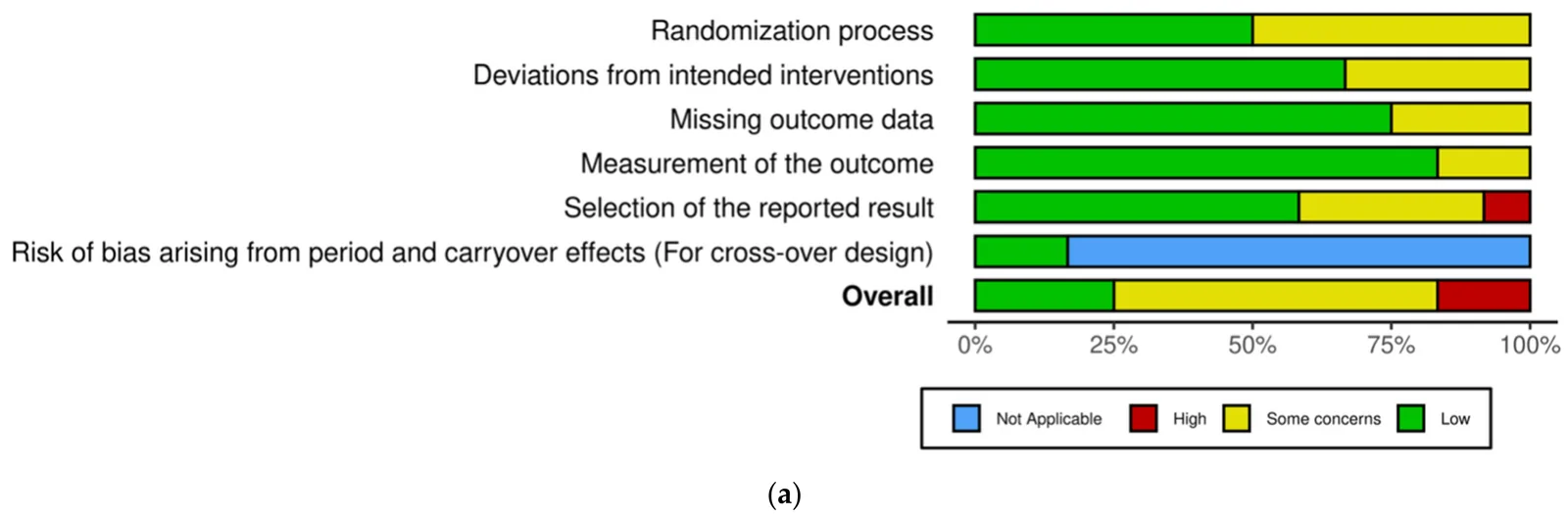

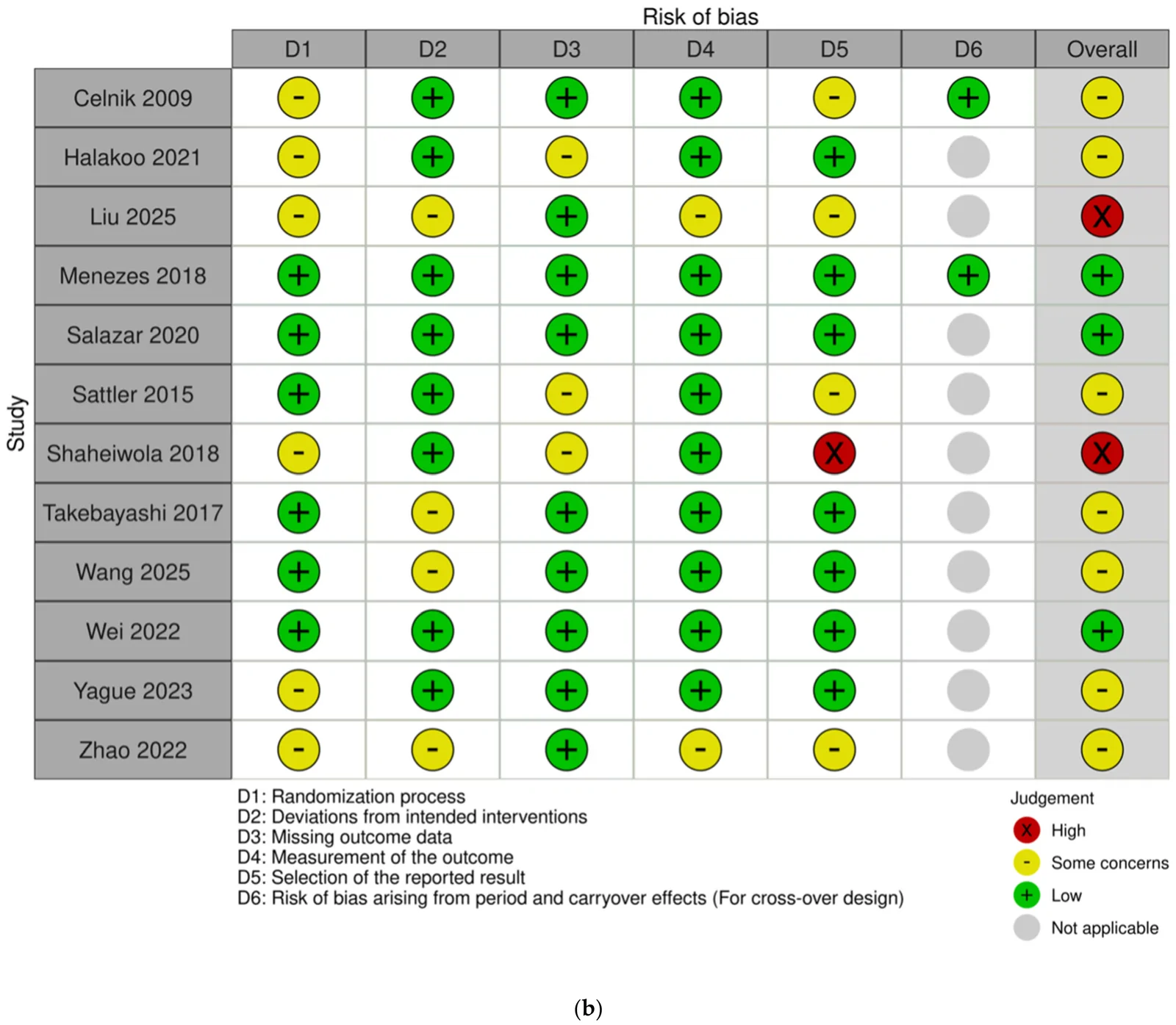
(**a**) Risk of bias graph for the included studies. (**b**) Risk of bias summary for the included studies [38,39,40,41,42,43,44,45,46,47,48,49].

The risk of bias (ROB) summary of the included studies is presented in Figure 2a,b. Overall, there was some concern about ROB in seven studies [40,41,43,44,45,47,49], while three studies [38,39,42] had a low risk and two studies [46,48] had a high risk of ROB. Allocation concealment was unclear in half of the included studies, which resulted in some concerns of ROB in the randomization process [44,45,46,47,48,49]. There were four studies that had some concerns of ROB due to deviation from the intended intervention because of a lack of subject and therapist blinding [41,43,48,49]. In total, three studies had some concern about ROB because of missing outcome data and a lack of intention-to-treat analysis [40,45,46]. Some concern about ROB in the measurement of outcomes due to a lack of assessor blinding was found in two studies [48,49]. Regarding the selection of reported results, there were some concerns of ROB in four studies due to an unclear pre-specified analysis plan [40,44,48,49], while one study had a high risk of ROB owing to a difference between the reported results and the pre-specified analysis and reporting plan [46]. The two crossover-designed studies had a low risk of ROB in carryover effects.

Although all the included RCTs demonstrated good-to-excellent quality in reporting completeness and statistical transparency according to the PEDro scale, high reporting compliance assessed by PEDro did not universally translate to a low risk of overall bias in internal validity under the Cochrane RoB 2 framework. Two studies [46,48] scored a good overall quality in PEDro yet had an overall high risk of bias according to the RoB 2 tool. Conversely, only three studies [38,39,42] achieved low risk of overall bias by the RoB 2 tool and good-to-excellent quality by the PEDro at the same time.

### 3.3. Study Characteristics

Table 2 presents the characteristics of the included studies. A more detailed table (Table S3) presenting the complete characteristics of the included studies can be found in Supplementary Materials Table S3.

#### 3.3.1. Participants

In total, 449 stroke patients participated in the 12 included studies, of whom 272 were male. The mean age of the participants ranged from 49.3 to 69.57 years. One study assessed stroke patients in the acute phase [40], two studies assessed the subacute phase [45], six studies assessed the chronic phase [38,39,41,42,44,46], and three studies assessed stroke phase varied from acute to chronic [43,47,49]. The mean stroke duration ranged from 5.3 ± 2.6 days to 5.7 ± 5.7 years. Regarding the stroke type, 250 participants had ischemic stroke, while 24 had hemorrhagic stroke. The stroke types of the remaining participants were not reported in four studies [40,45,46,48]. In total, 147 participants were affected on the left side, while 138 were affected on the right side. Four studies did not report the lesion side or affected side [40,45,47,48].

#### 3.3.2. Interventions

The 12 included studies applied heterogeneous tDCS and PES protocols (Table 3).

##### tDCS Parameters

Regarding the type of tDCS, anodal tDCS (a-tDCS) was applied by five studies [38,40,43,44,45], while bilateral-hemispheric tDCS (b-tDCS) was applied in seven studies [39,41,42,46,47,48,49]. All 12 included studies placed the anode electrode on the ipsilesional primary motor area (M1). A total of five studies applied the cathode electrode on the Contralateral supraorbital region [38,40,43,44,45], while the remaining seven studies applied the cathode electrode on the contralesional M1 [39,41,42,46,47,48,49]. Regarding the size of anode or active electrodes, one study reported 3 × 3 cm [44], two reported 5 × 7 cm [45,48], two reported 5 × 5 cm [39,46], one reported 35 cm^2^ [40], two reported 25 cm^2^ [42,47], and one reported 5 × 10 cm [43]. Three studies did not report the size of the electrodes [38,41,49]. Four studies applied an intensity of 1 mA [38,41,43,44], one study applied 1.2 mA [40], one study applied an intensity of 1.5 to 2 mA [48], and six studies applied an intensity of 2 mA [39,42,45,46,47,49]. Regarding tDCS duration, one study reported a duration of 13 min [40], eight studies reported 20 min [38,41,43,44,45,47,48,49], while two studies reported a duration of 30 min [39,42].

##### PES Parameters

Types of PES included peripheral nerve stimulation (PNS) in one study [44], functional electrical stimulation (FES) in five studies [39,45,46,48,49], repetitive peripheral nerve stimulation (RPNS) in two studies [40,47], neuromuscular electrical stimulation (NMES) [41,42], acupoint transcutaneous electrical nerve stimulation (Acu-TENS) in one study [43], and repetitive peripheral nerve sensory stimulation (RPSS) in one study [38].

All studies applied unilateral PES on the paretic side at the median nerve, ulnar nerve, radial nerve, extensor carpi radialis, extensor digitorum muscles, wrist extensor, extensor digitorum communis, anterior deltoid, serratus anterior, triceps brachii, wrist dorsal muscles, and acupoints of LI11, TE5, GB34, and ST37 of the paretic side. The stimulation frequency ranged from 5 to 100 Hz, and the pulse width ranged from 200 to 1000 µs. The duration of stimulation ranged from 10 to 120 min.

##### Sessions and Duration of tDCS Combined with PES

The number of sessions of tDCS combined with PES ranged from 4 to 20 sessions. Two studies conducted 4 sessions [38,44], two conducted 5 sessions [40,47], two conducted 10 sessions [39,45], two conducted 12 sessions [43,49], one conducted 7 to 14 sessions [48], one conducted 15 sessions [42], and two conducted 20 sessions [41,46]. Sessions lasted for a range of 1 to 8 weeks.

#### 3.3.3. Comparisons

Combined tDCS and PES was compared with active PES with/without sham tDCS (PES alone) in eight studies [38,39,40,44,45,46,47,49], active tDCS with/without sham PES (tDCS alone) in four studies [38,42,44,48], sham tDCS combined with sham PES in three studies [38,42,44], and standard therapy alone in 3 studies [41,43,48].

#### 3.3.4. Outcomes

##### Motor Function and Impairment

Ten studies reported upper limb motor function and impairment recovery. Five studies applied the Fugl–Meyer Assessment—Upper Extremity (FMA-UE) [39,41,42,46,47], three applied Fugl–Meyer Assessment—Wrist/Hand (FMA-WH) [40,48,49], one applied Fugl–Meyer Assessment—Upper Extremity and Lower Extremity (FMA-UE&LE) [43], and one reported the performance of the finger motor sequence task (FMST) [44].

##### Spasticity

Change in spasticity was reported in five studies using Modified Ashworth Scale (MAS) [42,43,45,46,47]. As the data provided by three studies were insufficient for meta-analysis [45,46,47], only two studies were involved in the quantitative synthesis of the MAS.

##### Strength

Two studies reported upper limb strength in terms of handgrip strength [39,40], while another one reported grasp strength and pinch strength [38].

##### Activities of Daily Living (ADL) Independence

Three studies evaluated activities of daily living (ADL) by Modified Barthel Index (MBI) [42,43,48].

##### Real-World Upper Limb Performance

One study evaluated real-world upper limb performance by assessing the amount of use (AOU) and quality of movement (QOM) by the Motor Activity Log (MAL) [41].

##### Upper Limb-Specific Activity Capacity

One study reported upper limb-specific activity capacity by the Wolf Motor Function Test (WMFT) [46], one study evaluated by the Jebsen and Taylor Hand Function Test (JTT) [40], while another one reported by the Action Research Arm Test (ARAT) [42].

#### 3.3.5. Study Design

All included studies were randomized controlled trials (RCTs). Except for two crossover-design studies [38,44], all studies had a parallel design.

### 3.4. Qualitative Synthesis and Quantitative Synthesis

#### 3.4.1. Effects of tDCS + PES Versus tDCS with/Without Sham PES (tDCS Alone)

##### Effects of tDCS + Motor-Level PES Versus tDCS Alone

**Figure 3:**
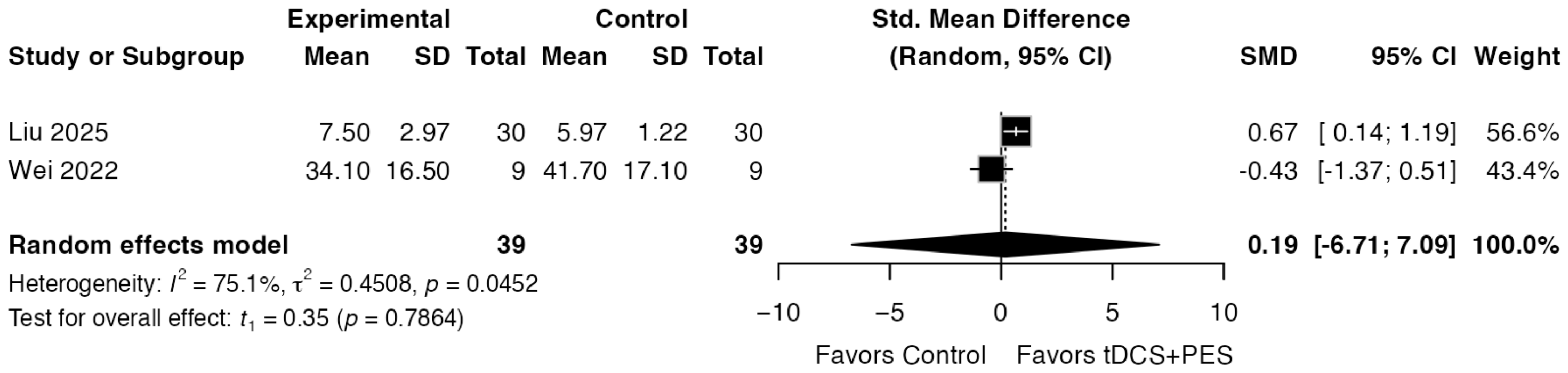
(Exploratory) Meta-analysis of the effects of tDCS + motor PES versus tDCS alone on FMA at post-intervention [42,48].

When compared with tDCS alone, tDCS combined with motor-level PES showed no significant effect on FMA-UE (MD = −7.60; 95% CI = −23.12 to 7.92; *p* = 0.34) in one study [42], while it showed a significant effect on FMA-WH (MD = 1.53; 95% CI = 0.38 to 2.68; *p* = 0.009) in the other study [48]. The two studies [42,48] were pooled for an exploratory meta-analysis across FMA variations (FMA-UE and FMA-WH) at post-intervention (Figure 3). The meta-analysis showed that tDCS combined with motor-level PES demonstrated no significant global FMA improvement (SMD = 0.19; 95% CI = −6.71 to 7.09; *p* = 0.79), with substantial heterogeneity (I^2^ = 75%) and very low certainty of evidence (Table 4).

One study [42] reported a non-significant effect on MAS reduction when comparing tDCS with motor-level PES to tDCS with sham PES (MD = −0.10; 95% CI = −0.93 to 0.73; *p* = 0.81).

**Figure 4:**
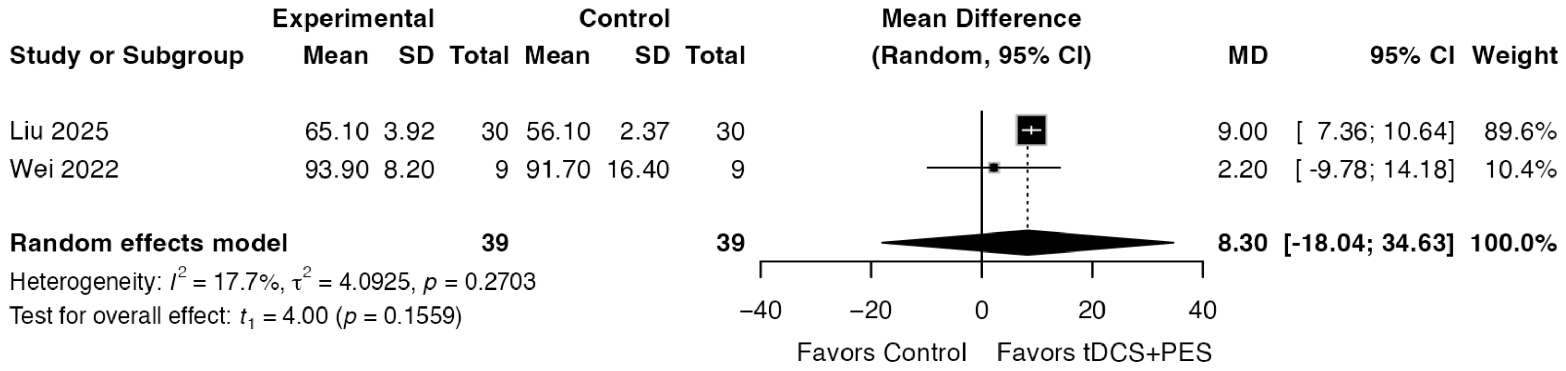
Meta-analysis of the effects of tDCS + motor PES versus tDCS alone on ADL at post-intervention [42,48].

Two studies [42,48] were pooled for the meta-analysis on ADL independence (MBI) at post-intervention (Figure 4). When compared with tDCS alone, pairing tDCS with motor-level PES demonstrated no significant improvement in ADL independence (MD = 8.30; 95% CI = −18.04 to 34.63; *p* = 0.16), with low heterogeneity (I^2^ = 18%) and low certainty of evidence (Table 4).

One study [42] reported no significant improvement in ARAT (MD = −9.80; 95% CI = −31.06 to 11.46; *p* = 0.37) when comparing tDCS with motor-level PES with tDCS with sham PES.

##### Effects of tDCS + Sensory-Level PES Versus tDCS Alone

One study [44] reported a 22.7% improvement in a finger motor sequence task at post-intervention when comparing tDCS with sensory-level PES to tDCS with sham PES.

#### 3.4.2. Effects of tDCS + PES Versus PES with/Without Sham tDCS (PES Alone)

**Figure 5:**
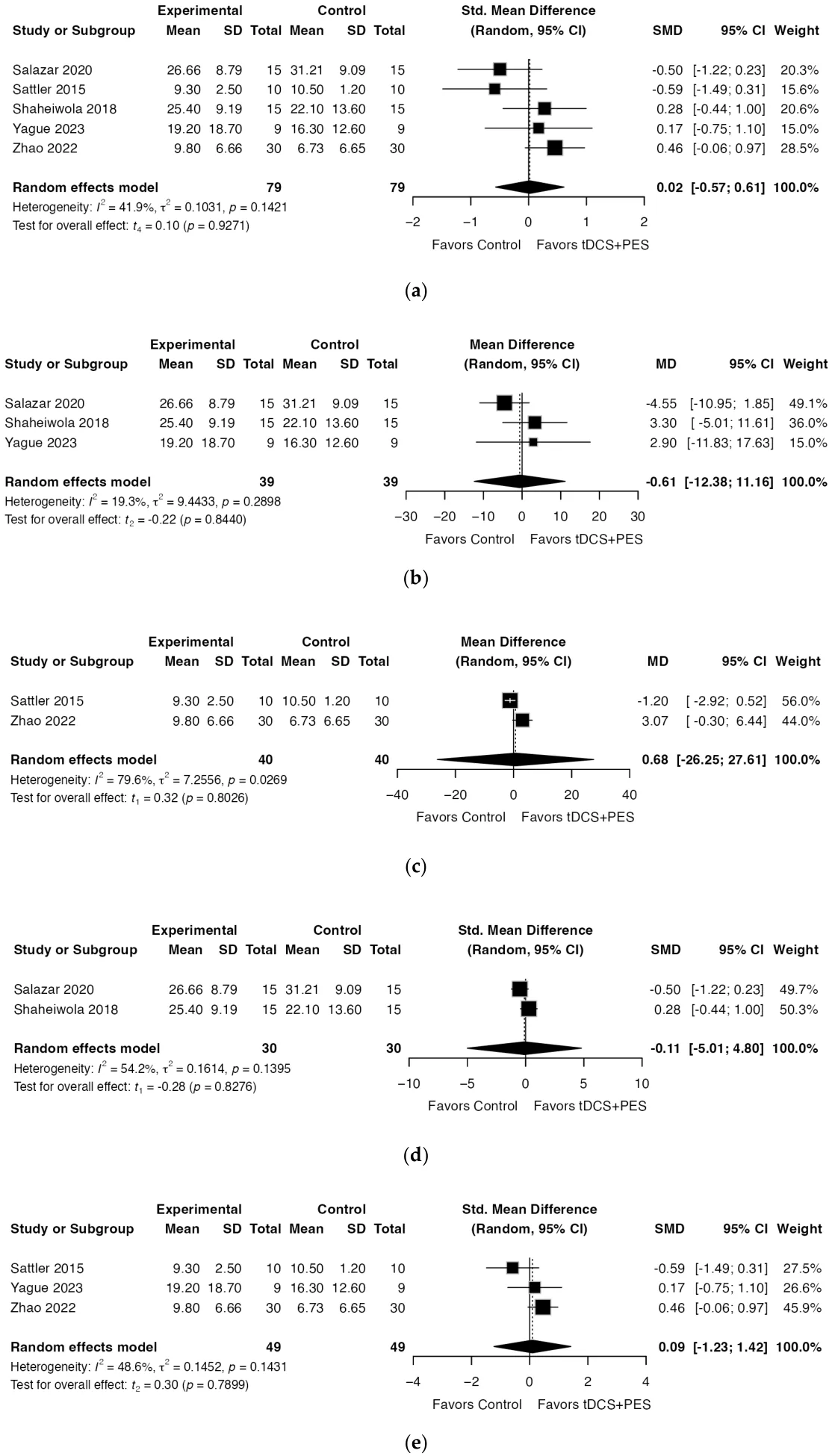
(**a**) (Exploratory) Meta-analysis on the effect of tDCS + motor/sensory PES versus PES alone on FMA (FMA-UE and FMA-WH) at post-intervention; (Exploratory) Subgroup analyses based on (**b**) FMA-UE; (**c**) FMA-WH; (**d**) chronic stroke phase; and (**e**) acute/subacute stroke phase [39,40,46,47,49].

An exploratory analysis on the pooled effect of combining tDCS with motor/sensory-level PES on FMA at post-intervention was conducted by pooling five studies [39,40,46,47,49]. It showed that tDCS combined with motor/sensory PES showed a non-significant overall effect on FMA when compared with PES alone (SMD = 0.02; 95% CI = −0.57 to 0.61; *p* = 0.93; I^2^ = 42%) (Figure 5a), with low certainty of evidence (Table 5). Subgroup analyses were conducted based on FMA variations (Figure 5b,c) and stroke phases (Figure 5d,e). Compared with PES alone, tDCS with motor/sensory PES demonstrated no significant effects on FMA in all subgroups, with a moderate to very low certainty (Table 5).

##### Effects of tDCS + Motor-Level PES Versus PES Alone

**Figure 6:**
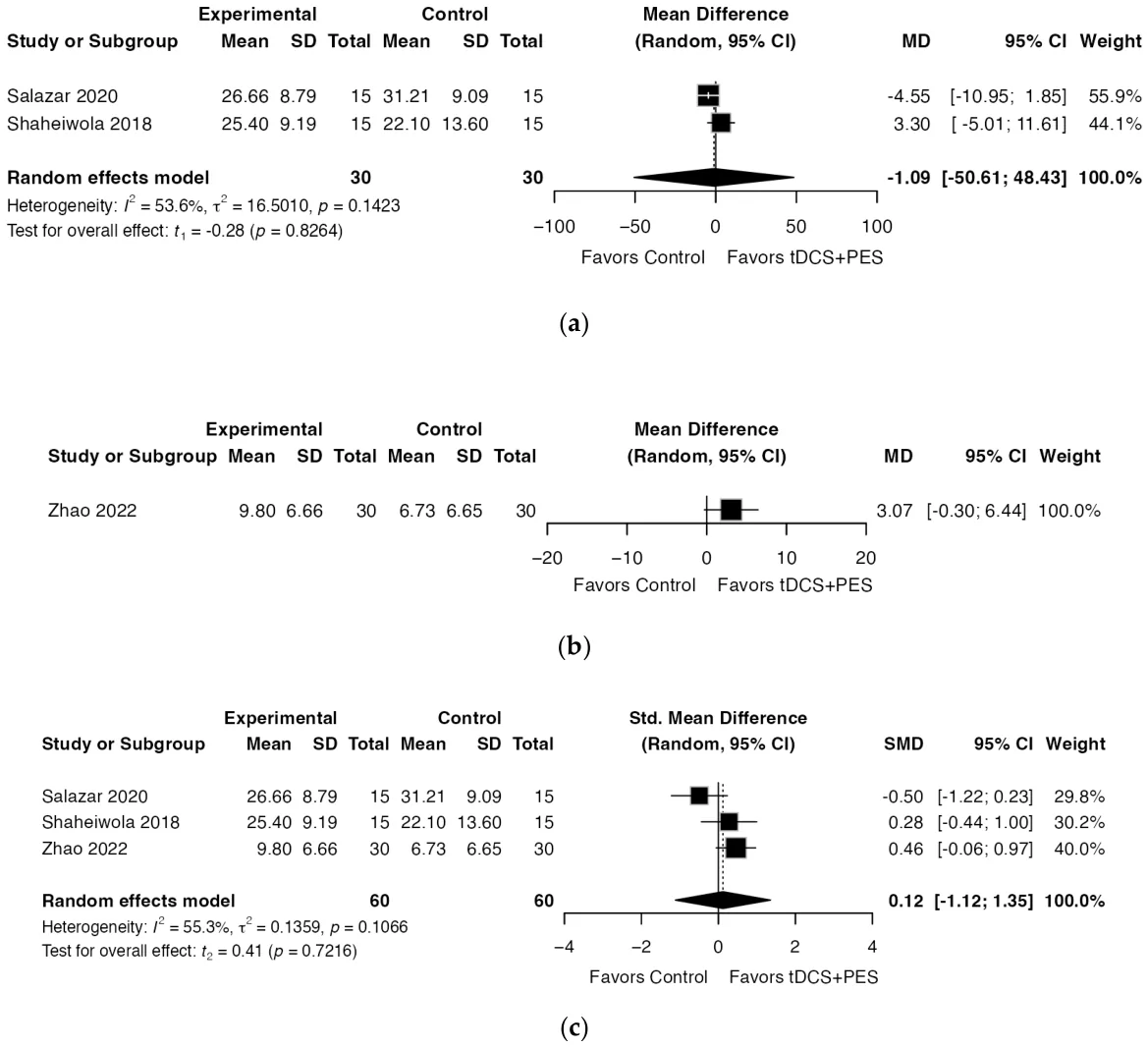
Subgroup analyses on the effect of tDCS + motor PES versus PES alone on (**a**) FMA-UE (in chronic stroke); (**b**) FMA-WH (in acute/subacute stroke) at post-intervention; (**c**) exploratory meta-analysis on the effect of tDCS + motor PES versus PES alone on FMA (FMA-UE and FMA-WH) at post-intervention [39,46,49].

When compared with PES alone, pairing tDCS with motor-level PES demonstrated no significant effects on FMA-UE in chronic stroke phase (MD = −1.09; 95% CI = −50.61 to 48.43; *p* = 0.83; I^2^ = 54%) (Figure 6a) [39,46], and FMA-WH in acute/subacute stroke phase (MD = 3.07; 95% CI = −0.30 to 6.44; *p* = 0.07) (Figure 6b) [49]. Exploratory meta-analysis across both FMA variations showed that tDCS combined with motor-level PES showed no overall significant effect on FMA (FMA-UE and FMA-WH) at post-intervention when compared with PES alone (SMD = 0.12; 95% CI = −1.12 to 1.35; *p* = 0.72; I^2^ = 55%) (Figure 6c), with very low certainty of evidence (Table 6) [39,46,49].

Despite incomplete data reported for meta-analysis and effect size estimation, two studies reported effects on MAS. One of them [45] indicated a significant MAS reduction; however, another study reported [46] no significant reduction in MAS when compared with PES alone at post-intervention.

One study [39] demonstrated a significant effect favoring tDCS with motor-level PES on handgrip strength when compared with PES alone at post-intervention.

When compared with sham tDCS with PES, one study reported a significant WMFT improvement at post-intervention [46].

##### Effects of tDCS + Sensory-Level PES Versus PES Alone

**Figure 7:**
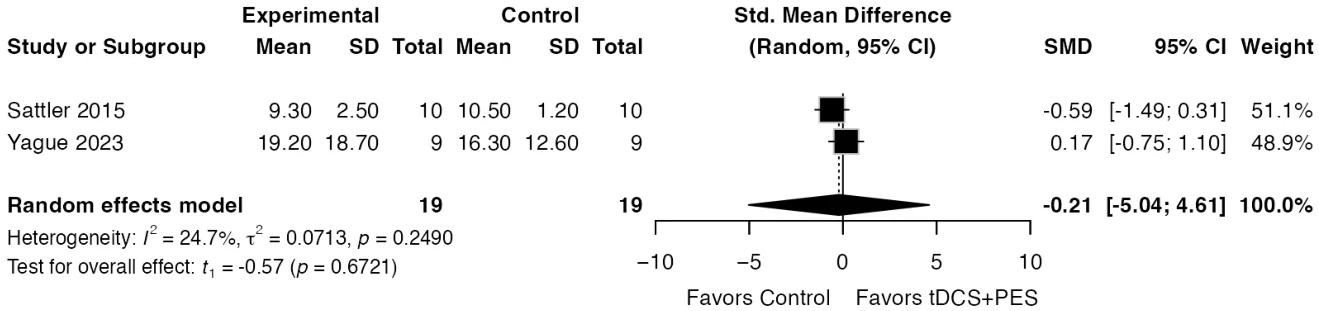
Exploratory meta-analysis on the effect of tDCS + sensory PES versus PES alone on FMA at post-intervention [40,47].

Compared with PES alone, pairing tDCS with sensory-level PES demonstrated no significant effects on both FMA-UE (MD = 2.90; 95% CI = −11.83 to 17.63; *p* = 0.70) [47], and FMA-WH (MD = −1.20; 95% CI = −2.92 to 0.52; *p* = 0.17) [40]. An exploratory meta-analysis across both FMA variations showed that tDCS with sensory PES yielded no overall significance on FMA improvement (SMD = −0.21; 95% CI = −5.04 to 4.61; *p* = 0.67; I^2^ = 25%) (Figure 7), with moderate certainty of evidence (Table 7) [40,47].

One study [44] reported a better performance (15.4%) in the finger motor sequence task at post-intervention when comparing tDCS with sensory PES to sham tDCS with PES.

Compared with PES alone, pairing tDCS with sensory-PES showed no significant MAS reduction at post-intervention in one study [47].

Compared with PES alone, no significant effects on handgrip [40], grasp, and pinch strength [38] were observed when pairing tDCS with sensory-level PES.

A non-significant effect on JTT at post-intervention was reported by one study when adding tDCS to sensory-level PES [40].

#### 3.4.3. Effects of tDCS + PES Versus Sham tDCS + Sham PES

Three studies [38,42,44] involving 46 participants evaluated outcomes by distinct and non-overlapping assessment scales. Therefore, a meta-analysis comparing dual-stimulation and sham dual-stimulation could not be conducted.

##### tDCS + Motor-Level PES Versus Sham tDCS + Sham PES

In one study [42], significant post-intervention improvements in FMA-UE, ARAT, MAS, and MBI were observed in the tDCS with the motor-level PES group, whereas the sham group showed no significant effects. However, between-group comparisons did not reach significance in all four outcomes.

##### tDCS + Sensory-Level PES Versus Sham tDCS + Sham PES

One study [44] reported a 41.3% improvement in the finger motor sequence task at post-intervention when comparing tDCS with sensory-level PES to the sham group.

In addition, no significant effects on grasp and pinch strength favoring tDCS with sensory-level PES were observed [38].

#### 3.4.4. Effects of tDCS + PES Versus Standard Therapy Alone

**Figure 8:**
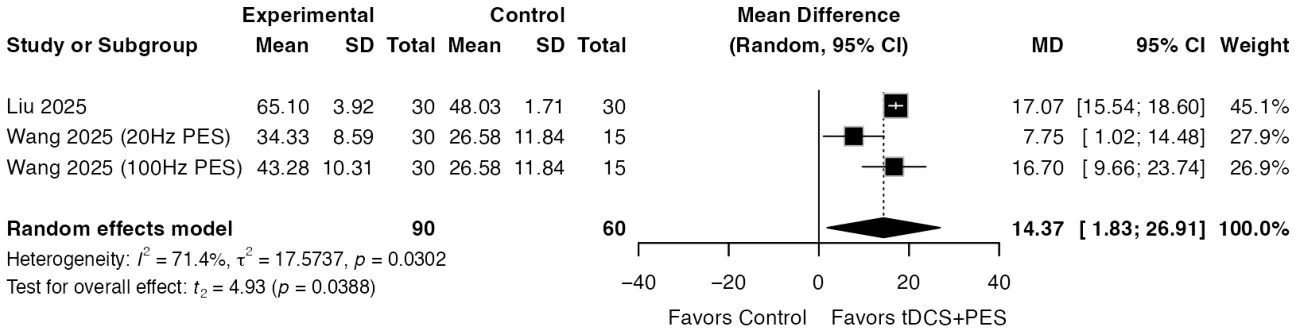
(Exploratory) Meta-analysis of the effects of tDCS + motor/sensory PES versus standard therapy alone on ADL at post-intervention (with very low certainty of evidence; substantial heterogeneity, I^2^ = 71%; and heavily weighted by a study [48], 45.1%) [43,48].

Two studies [43,48] with 150 participants were pooled for the meta-analysis on ADL independence at post-intervention (Figure 8). When compared with standard therapy alone, tDCS with motor/sensory-level PES showed a significant improvement in ADL (assessed by MBI) (MD = 14.37; 95% CI = 1.83 to 26.91; *p* = 0.039), with substantial heterogeneity (I^2^ = 71%) and very low certainty of evidence (Table 8).

##### tDCS + Motor-Level PES Versus Standard Therapy Alone

When compared with standard therapy alone, combining tDCS and motor-level PES with standard therapy demonstrated no significant benefit on FMA-UE (MD = 2.20; 95% CI = −5.52 to 9.92; *p* = 0.58) in one study [41], while a significant benefit on FMA-WH (MD = 5.17; 95% CI = 4.09 to 6.25; *p* < 0.001) was reported in another study [48].

Subgroup analysis showed that tDCS combined with motor-level PES demonstrated a significant ADL improvement at post-intervention (MD = 17.07; 95% CI = 15.54 to 18.60; *p* < 0.001) when compared with standard therapy alone in one study [48].

Another study [41] reported that tDCS combined with motor-level PES demonstrated a significant effect on MAL-AOU, but not on MAL-QOM relative to CIMT alone.

##### tDCS + Sensory-Level PES Versus Standard Therapy Alone

**Figure 9:**
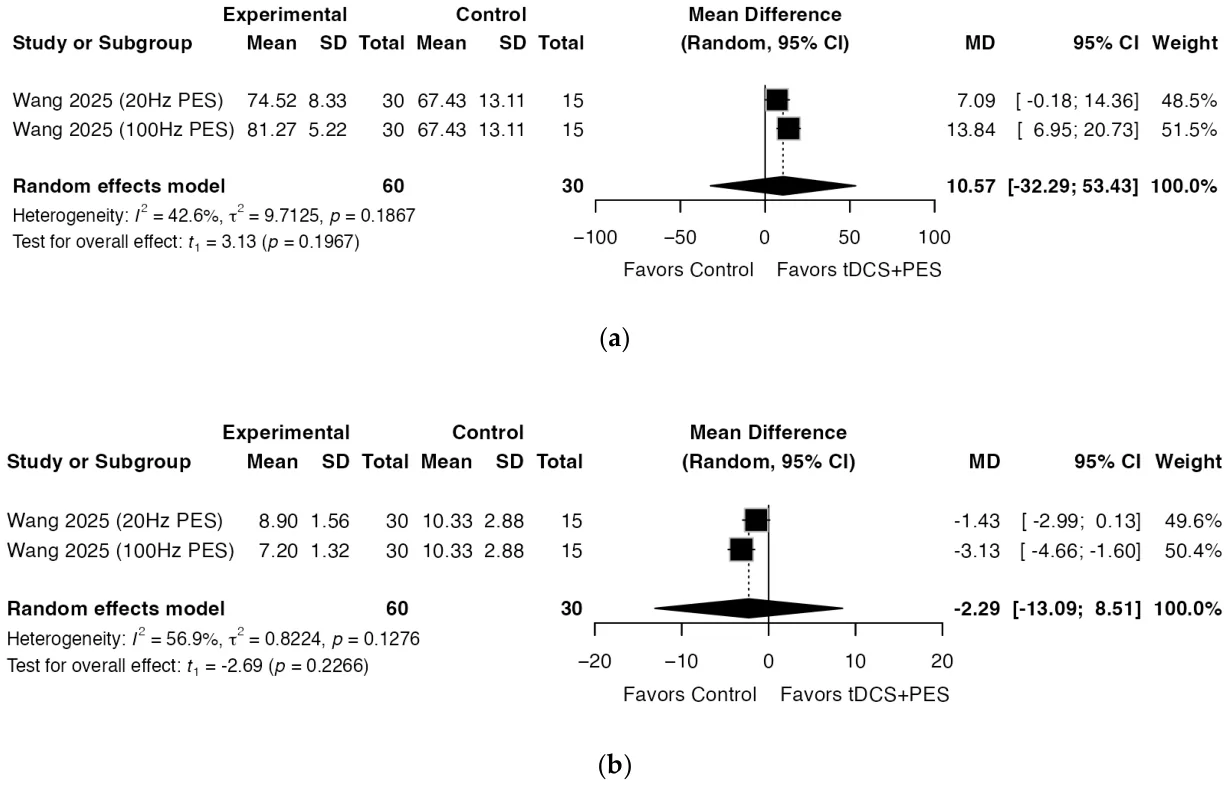

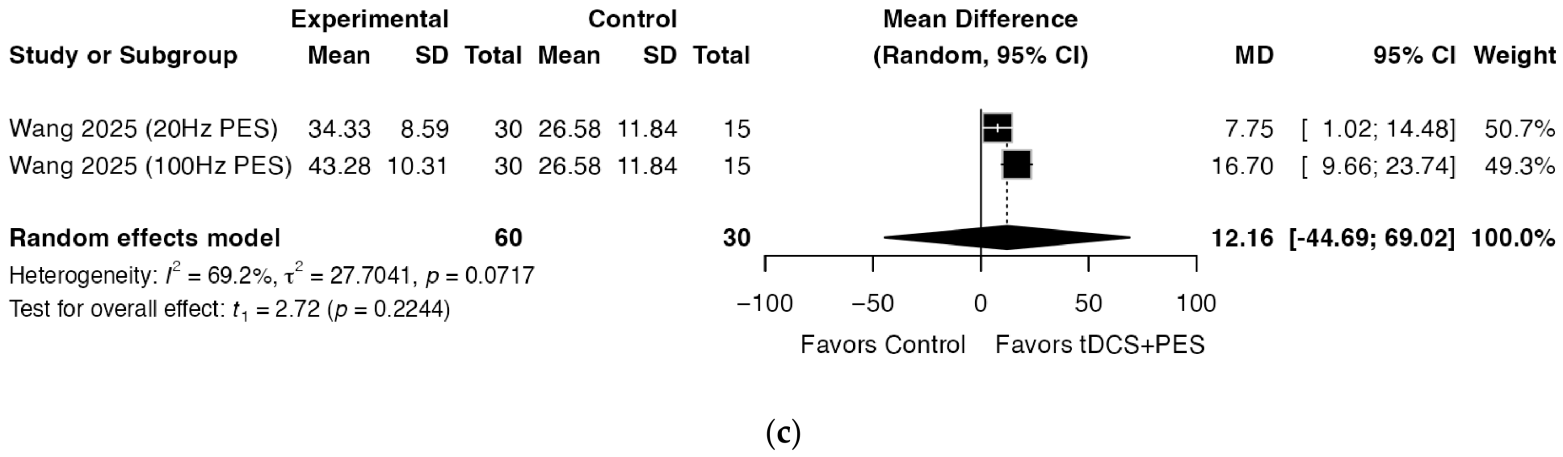
(Exploratory) Meta-analysis of the effects of tDCS + sensory PES versus standard therapy alone on (**a**) FMA-UE&LE, (**b**) MAS, and (**c**) ADL at post-intervention [43].

A study [43] compared tDCS combined with sensory-level PES with different frequencies (20 Hz/100 Hz) to standard care alone. When compared with standard care alone, the group that applied 100 Hz sensory-level PES demonstrated significant effects on FMA-UE&LE, MAS, and MBI, while the group of 20 Hz showed non-significant effects. Exploratory meta-analyses were conducted by pooling the two comparisons from the same study (tDCS + 20 Hz Acu-TENS/tDCS + 100 Hz Acu-TENS vs. standard care alone) (Figure 9). By pooling the two comparisons from the same study [43], combining tDCS and sensory-level PES with standard therapy showed no significant overall improvement in FMA-UE&LE (MD = 10.57; 95% CI = −32.29 to 53.43; *p* = 0.197, I^2^ = 43%) (Figure 9a), MAS reduction (MD = −2.29; 95% CI = −13.09 to 8.51; *p* = 0.227; I^2^ = 57%) (Figure 9b), and ADL improvement (MD = 12.16; 95% CI = −44.69 to 69.02; *p* = 0.224; I^2^ = 69%) (Figure 9c), with low certainty of evidence (Table 8).

### 3.5. Results of Sensitivity Analysis

#### 3.5.1. Sensitivity Analysis by “Leave-One-Out”

Sensitivity analyses were performed by “leave-one-out” criteria (Table 9). Compared with PES alone, sensitivity analyses detected minimal changes in the effects on FMA by tDCS combined with motor PES and tDCS combined with motor/sensory PES, which remained non-significant. Compared with standard therapy alone, the significant effect on ADL improvement by tDCS combined with motor/sensory PES was reduced to non-significant levels when excluding two arms [43,48] separately.

#### 3.5.2. Sensitivity Analysis by DL Random-Effects Model via RevMan

Sensitivity analyses were performed by applying the standard DerSimonian–Laird (DL) random-effects model via RevMan (Table 10). Compared with standard therapy alone, the ADL improvement by tDCS combined with motor/sensory PES remained significant in both analyses (*p* = 0.039 by REML + HK via R and *p* < 0.001 by DL via RevMan).

## 4. Discussion

In this study, a systematic review and meta-analysis of the effect of combined tDCS and PES on upper limb motor function after stroke was conducted. It included 12 RCTs. Qualitative narrative synthesis and quantitative synthesis were used for data analysis.

**Effects of combined tDCS and motor-level PES over individual stimulation alone**

Current evidence does not demonstrate a clear synergistic or additive effect of pairing tDCS and motor-level PES on upper limb function recovery after stroke. Compared with tDCS alone, adding motor-level PES to tDCS showed no significant additive benefits on FMA-UE, spasticity, ADL independence, and activity capacity. In addition, when compared with PES alone, adding tDCS to motor-level PES showed no significant effects on FMA-UE and FMA-WH. These challenge the hypothesized synergistic or additive effect on upper limb function of combining tDCS with motor-level PES. It suggests that when peripheral pathways are already actively stimulated via motor-level PES, adding tDCS may provide negligible further benefit to upper limb motor function. According to qualitative synthesis, when compared with tDCS alone or PES alone, apart from one study [45], another two studies [42,46] reported no significant MAS reduction narratively. Such conflicting results suggest that pairing tDCS and motor-level PES may not generally outperform individual stimulation on upper limb anti-spasticity. More future RCTs are needed to verify the upper limb anti-spasticity effects of dual stimulation and individual stimulation.

Although possible significant benefits on FMA-WH [48] by adding motor-level PES to tDCS and handgrip strength [39] as well as upper limb-specific activity capacity [46] by adding tDCS to motor-level PES were reported, they came from individual studies separately. Therefore, such potential additive benefits in wrist and hand function, handgrip strength, and upper limb-specific activity capacity are yet to be confirmed and should be further investigated in future studies.

**Effects of combined tDCS and motor-level PES over standard therapy alone**

At an individual study level, qualitative synthesis revealed some isolated therapeutic effects on upper limb function when adding tDCS and motor-level PES to standard rehabilitation therapy. The reported improvements in FMA-WH and upper limb performance align with a previous study [50]. However, since these findings are derived from isolated studies, they represent changes from small-sample cohorts only and may be attributed to study-level risk of bias. Meanwhile, by considering the non-significant effects on FMA-UE reported [41,42], the overall findings do not indicate a generalized beneficial effect on upper limb motor function after stroke by adding tDCS and motor-level PES to standard rehabilitation therapy.

**Effects of combined tDCS and sensory-level PES over individual stimulation alone**

Current evidence does not support the hypothesized central–peripheral synergy of combining tDCS with sensory-level PES on upper limb function recovery after stroke. Apart from one study which reported a possible benefit on finger function [44], the current quantitative and qualitative synthesis showed no significant synergistic or additive benefits on upper limb motor function, spasticity, muscle strength, and activity capacity when pairing tDCS with sensory-level PES.

**Effects of combined tDCS and sensory-level PES over standard therapy alone**

The overall findings showed no clear beneficial effect on upper limb function after stroke when pairing tDCS and sensory-level PES with standard rehabilitation therapy. In general, the included studies reported null effects on upper limb motor function, spasticity, ADL, and muscle strength when adding tDCS and sensory-level PES to standard therapy. Although possible significant beneficial effects on upper limb motor function, spasticity, and ADL were shown when pairing tDCS with sensory-level PES at higher intensity (100 Hz), such potential effects were reported by a single study only [43]. Future RCTs pairing tDCS with high-intensity sensory-level PES are needed to examine and verify such intensity-dependent potential effects on upper limb function.

**Effects of combined tDCS and motor/sensory-level PES over standard therapy alone**

In addition, exploratory analysis revealed that combining tDCS and either motor-level or sensory-level PES with standard therapy demonstrated a significantly better ADL independence (with very low certainty of evidence). It suggested a potential value of adding tDCS and PES to standard therapy and that tDCS combined with PES may enhance the impact of standard therapy on ADL independence after stroke. However, as this pooled effect was heavily driven by one study (weighted 45.1%) [48] and was substantially heterogeneous (I^2^ = 71%) due to differences in PES modalities (CCFES and Acu-TENS), such possible ADL improvement by pairing tDCS and PES with standard therapy is fragile, with very low certainty of evidence, that must not be regarded as strong evidence of effectiveness yet. In addition, when excluding the arm applying low-intensity (20 Hz) PES from Wang (2025) [43], the sensitivity analysis, including two studies applying PES at higher intensity (60 Hz and 100 Hz), showed a more significant effect size with no heterogeneity (MD = 17.05; 95% CI = 16.08 to 18.03; *p* = 0.003; I^2^ = 0%) [43,48]. It suggested that the potential ADL improvement of pairing tDCS and PES with standard therapy may be driven by the stimulation intensity of PES. Future factorial RCTs are required to further verify such a potential effect of adding tDCS and PES to standard rehabilitation therapy on ADL after stroke.

**Clinical importance**

FMA-UE primarily evaluates proximal motor function, including reflex activities and gross movement synergies of the shoulder, elbow, and forearm. The minimum clinically important difference (MCID) for FMA-UE in stroke patients was estimated to be 4 to 12.4 points [51,52,53,54]. tDCS combined with motor-level PES yielded an MD of −7.60 (95% CI = −23.12 to 7.92; *p* = 0.34), −1.09 (95% CI = −50.61 to 48.43; *p* = 0.83), and 2.20 (95% CI = −5.52 to 9.92; *p* = 0.58) points on FMA-UE when compared with tDCS alone, PES alone, and standard therapy alone, respectively. In addition, tDCS combined with sensory-level PES demonstrated an MD of 2.90 (95% CI = −11.83 to 17.63; *p* = 0.70) points on FMA-UE when compared with PES alone. These findings indicated a lack of clinical importance of upper limb proximal motor function recovery after stroke when referenced to MCID. As the effects are highly imprecise and the 95% CIs are extremely wide, the overall clinical importance of upper limb proximal motor function recovery after stroke is highly uncertain. It suggests that tDCS combined with PES may not demonstrate a clinical advantage on FMA-UE over tDCS alone, PES alone, or standard therapy alone.

On the other hand, FMA-WH isolates distal fine-motor function, specifically evaluating wrist stability, grasp configurations, and finger dexterity. The MCID for FMA-WH in stroke patients was estimated to be 4 points [51]. tDCS combined with motor-level PES yielded an MD of 3.07 (95% CI = −0.30 to 6.44; *p* = 0.07) points on FMA-WH, while tDCS combined with sensory-level PES demonstrated an MD of −1.20 (95% CI = −2.92 to 0.52; *p* = 0.17) on FMA-WH when compared with PES alone. It indicates that any clinically meaningful advantage in distal dexterity function by the combined stimulations over PES alone is not demonstrated, as neither MD meets the MCID threshold.

In addition, when compared with standard therapy alone, tDCS paired with motor-level PES yielded an MD of 5.17 (95% CI = 4.09 to 6.25; *p* < 0.001) points on FMA-WH that exceeds the MCID threshold. Although this suggests a possible clinical advantage in upper limb distal motor function by combining tDCS and motor-level PES over standard therapy alone, such clinical importance is yet to be concluded as it was reported by an individual study only.

In addition, the MCID for MAS in the stroke population was estimated to be −0.48 points [55]. When compared with standard therapy alone, tDCS combined with sensory-level PES showed an MD of −2.29 points (95% CI = −13.09 to 8.51; *p* = 0.227). Although such MD exceeds the MCID, it was basically based on two comparisons from a single study consisting of distinct PES intensities (20 Hz/100 Hz). Due to the limited number of included arms, this finding indicates that while clinically meaningful benefit on spasticity by adding tDCS and sensory-level PES to standard rehabilitation therapy is possible, the current evidence remains exploratory.

Furthermore, the MCID for MBI in stroke patients was estimated to be 4 to 5 points [56]. When comparing with tDCS alone, tDCS combined with motor-level PES yielded an MD of 8.30 points on MBI (95% CI = −18.04 to 34.63; *p* = 0.16; I^2^ = 18%). Although the effect size meets the MCID threshold, its 95% CIs are extremely wide and it was largely contributed by one study (weighted 89.6%). Therefore, any clinical advantage of adding motor-level PES to tDCS on MBI is conservative.

Exploratory meta-analysis, evaluating the combined stimulation against standard therapy alone, yielded an MD of 14.37 on MBI (95% CI = 1.83 to 26.91; *p* = 0.039; I^2^ = 71%), which demonstrated a clinical advantage. Although the 95% CI reflected imprecision by spanning widely, it still demonstrated clinical benefit as its lower boundary still exceeds the MCID threshold. However, since this effect had a very low certainty of evidence and substantial heterogeneity (I^2^ = 71%) due to the inclusion of different PES modalities (both motor-level and sensory-level PES) and heavy weighting contributed by a single study, this clinically meaningful benefit on MBI is preliminary and remains uncertain. Future validation of this potential clinical benefit on MBI is required.

**Current findings and future directions**

Despite the theoretical hypothesis of synergistic or additive effects of central–peripheral stimulations through mechanisms of associative plasticity [15,21], the absence of significant effects on upper limb function when compared with tDCS alone and PES alone did not demonstrate clear support for the hypothesized synergistic or additive effect. As neuromodulatory effects are state-dependent and non-linear, the overall inconsistent pooled effects could be a result of variable effects canceling out each other when applying similar stimulations to RCTs with heterogeneous stroke populations. The absence of a clear synergistic or additive effect of pairing tDCS and PES on upper limb function after stroke in our current evidence may be due to the various state-dependent variability, lesion characteristics, stimulation parameters and timing, heterogeneous stimulation protocol, and patient responsiveness across RCTs.

Moreover, although tDCS has been hypothesized to play a dominant therapeutic role while PES serves as a complementary stimulus [15], our findings lack consistent evidence to validate such mechanistic interaction. The potential overall improvement in ADL independence, when compared with standard therapy alone, reflected the possible benefit of adding multimodal stimulation to rehabilitation therapy, but the mechanistic interaction between central top-down and peripheral bottom-up inputs remains complex and unverified by current evidence. Previous reviews examining tDCS or PES have reported modest improvements in motor function and structure domain [20,57]. Our findings suggested that adding both tDCS and PES to rehabilitation programs appears to be potentially beneficial for activity-level functional independence (e.g., ADL) in stroke rehabilitation. However, such a beneficial effect did not uniformly extend to the broader upper limb function and structure domain. Our findings extend the existing literature by suggesting that activity and participation domains may be more responsive to dual central–peripheral stimulation. Given the high variability in intervention protocols and low certainty of evidence, future studies must standardize stimulation protocols to verify these domain-specific differences.

In addition, the hypothesized combined central–peripheral paradigms may be evaluated alongside established associative stimulation protocols, such as paired associative stimulation (PAS). PAS pairs transcranial magnetic stimulation (TMS) over M1 with peripheral nerve stimulation at precise inter-stimulus intervals to induce Hebbian spike-timing-dependent plasticity (STDP). Previous studies demonstrated that PAS effectively induces LTP- or LTD-like corticospinal excitability changes in both healthy individuals and stroke survivors [58,59,60,61], with novel variations, like visuomotor, highlighting how multimodal sensory-motor integration may significantly boost corticospinal output after stroke [60]. However, fundamental physiological differences exist between the concurrent tDCS and PES paradigms and PAS. While PAS relies on millisecond-level temporal precision to synchronize peripheral afferent signals with central magnetic pulses to trigger STDP, tDCS combined with PES generally utilizes continuous and subthreshold direct current via tDCS alongside tonic or burst PES. Rather than driving strict Hebbian associative plasticity via millisecond timing, tDCS combined with PES relies on sustained tonic priming of baseline cortical excitability and continuous somatosensory feedback. The lack of clear synergistic or additive efficacy observed in our findings may be due to the non-linear and state-dependent nature of continuous direct current stimulation. Unlike discrete, timed-pulse protocols via PAS, continuous dual stimulation via tDCS and PES may induce homeostatic metaplasticity or ceiling effects that neutralize potential additive benefits. Future studies should explore whether structured temporal coupling or pulse-synchronized central–peripheral protocols, similar to PAS principles, can better optimize upper limb motor recovery in stroke populations.

To determine whether combined tDCS and PES are truly synergistic, additive, or simply independently effective to upper limb function after stroke, future studies should employ factorial designs that include at least the four arms of combined tDCS and PES, tDCS alone, PES alone, and sham or standard therapy control. Such a factorial design is methodologically crucial to differentiate whether the effects of tDCS with PES represent the hypothesized neuroplastic synergy, a simple additive effect, or a nonspecific effect. Exploring these mechanisms through factorial protocols alongside standardized interventional protocols, such as uniform stimulation intensity, frequency, sites, dosage, etc., is essential to advance the mechanistic clinical translation in post-stroke neurorehabilitation.

**Heterogeneity and subgroup analysis**

The overall null findings on upper limb motor function, such as FMA-UE, may reflect the limitations of evaluating multimodal protocols through a simple additive mechanism. From a state-dependent and network-level plasticity perspective, peripheral input acts as a dynamic modulator of the central cortical network, where therapeutic efficacy relies on the dynamic alignment between ascending somatosensory afferent impulses and descending corticospinal excitability. For example, if the patient’s baseline cortical excitability, interhemispheric inhibitory balance, or corticospinal tract (CST) integrity is highly compromised, the sensorimotor network may lack the neural architecture required to integrate paired central and peripheral inputs effectively. Thus, state-dependent variability across stroke populations may yield different effects from the same dual stimulation. Furthermore, timing and task-dependent variables, such as whether tDCS and PES are delivered simultaneously at rest or paired concurrently during motor training, may shift the cortical network into fundamentally different processing modes. So, differences in timing and synchrony may also induce state-dependent variables that affect the impact of the combined stimulation across different stroke populations. Therefore, applying uniform tDCS and PES parameters across heterogeneous stroke cohorts may produce variable effects that eventually cancel each other’s effects in pooled analyses. It reflects that the absence of a clear pooled effect may not reflect a complete failure of the hypothesized central–peripheral synergy but rather underscores that the therapeutic efficacy of pairing tDCS and PES may depend on state-dependent variability, such as baseline cortical excitability and network-level neuroplasticity, structural lesion characteristics, stimulation parameters and protocol, temporal timing and synchrony, and individual patient responsiveness. Future RCTs should adopt state-dependent stratification protocols by systematically stratifying stroke populations according to their state-dependent variables and precisely coupling stimulations with aligned timing, parameters, and protocol.

The moderate to high heterogeneity may be a result of the diverse interventional landscape across studies, such as tDCS montage, PES modalities, stimulation intensity, frequencies, pulse widths, and treatment dosages, etc. Apart from the factors in the subgroup analyses we conducted, several other clinical moderators may have accounted for the heterogeneity. While our exploratory subgroup analyses on FMA stratified the data by FMA subscales, sensory/motor PES, and stroke phases, significant heterogeneity persisted in several subgroups (80% in FMA-WH; 55% in motor PES; 54% in chronic stroke; 49% in acute/subacute stroke). This indicated that single-factor clinical stratification is insufficient to completely resolve the multi-dimensional variance across studies. Firstly, as electrode configurations of tDCS alternated between ipsilesional anodal excitation and bihemispheric modulation, various tDCS configurations contributed to the heterogeneity. By excluding the only study that applied anodal tDCS [40], the rest of the included studies applying bihemispheric tDCS yielded a reduced heterogeneity (I^2^ reduced from 42% to 33%) in our sensitivity analysis. However, due to the various current intensities (ranging from 1 to 2 mA) and stimulation durations (spanning from 13 to 30 min), the diversity of tDCS parameters across RCTs still contributed to the notable heterogeneity in our quantitative findings. Secondly, different PES parameters across studies, such as different PES modalities (FES vs. RPNS), stimulation frequency (ranging from 5 to 100 Hz), intensity, and dosage, also led to statistical variance. Thirdly, inconsistent stimulation dosages (ranging from 4 to 20 sessions that lasted for 5 days to 8 weeks) also led to heterogeneity. So, although distinct comparator types were stratified (namely the active tDCS alone, active PES alone, dual sham, and standard therapy alone), residual variations in the tDCS and PES protocols, combination of different types of tDCS and PES, types of conventional rehabilitation, etc., contributed to the notable heterogeneity in our quantitative findings.

Crucially, “tDCS combined with PES” should not be conceptualized as a single, uniform intervention. From a neurophysiological perspective, the specific combination of parameters dictates the nature of the central–peripheral stimulation. For instance, anodal tDCS primarily facilitates cortical excitability via neuronal depolarization, whereas cathodal montages induce hyperpolarization [62]. Combining distinct central stimulation with varying PES modalities may lead to distinct neuromodulatory mechanisms. For example, the mechanisms of pairing tDCS with sensory stimulation (that drives long-term potentiation-like plasticity) or motor stimulation (that directly evokes muscle contractions) are different. Nonetheless, the variations in stimulation intensity and overall treatment dosage (including the number of sessions and duration) further introduced dose–response variables that obscure the certainty of evidence in our meta-analyses. Due to the limited number of studies and small sample sizes per parameter, we were unable to conduct more meaningful subgroup analyses to explore the effect of different parameters separately. Consequently, the statistical findings of meta-analyses are regarded as an aggregate of diverse tDCS and PES protocols rather than the effects of a singular standardized therapy. This heterogeneity highlights the critical need for future optimization studies to identify the precise parameters of tDCS montages and PES modalities that maximize the therapeutic effect.

The marked heterogeneity and incomplete parameter reporting observed across the included RCTs reflect an urgent need for standardized reporting guide in dual-stimulation neuromodulation research. To optimize reproducible research protocols, facilitate precise cross-study comparisons, and advance clinical translation, we propose a minimum reporting standard for combined tDCS and PES for stroke rehabilitation in future studies as presented in Table 11.

**Methodological quality and risk of bias considerations**

Although the overall PEDro metrics categorized studies as possessing “good” to “excellent” methodological quality, some items revealed critical vulnerabilities. None of the 12 included studies achieved therapist blinding due to the inherent logistical difficulties of delivering active and sham neuromodulatory setups when blinding the therapist. Furthermore, allocation concealment was completely unfulfilled or poorly reported in half of the included studies [44,45,46,47,48,49], introducing a structural susceptibility to selection bias during randomization. In addition, our RoB 2 assessments showed that high-quality ratings on PEDro scale do not necessarily preclude critical risks of bias. For instance, only 3 [38,39,42] out of 12 RCTs demonstrated an overall low risk of bias, while the majority had some concern or high risk of bias. The domains driving these concerns include prominent deviations from intended interventions in four studies [41,43,48,49] due to unblinded personnel, missing outcome data coupled with a lack of intention-to-treat analysis in three studies [40,45,46], and selective reporting or unclear pre-specified analysis frameworks in five studies [40,44,46,48,49]. These domain-specific biases lead to a conservative certainty of evidence.

The structural limitations identified across ROB domains downgraded the certainty of the pooled non-significance on FMA when compared with PES alone. As this pooled result was synthesized from RCTs where three out of five failed to verify allocation concealment [44,45,46,47,48,49], it introduced selection bias that affected the overall certainty of evidence and obscured the true effects.

In addition, the GRADE certainty of evidence was consistently downgraded to “moderate” to “very low”. Firstly, behavioral and activity-based outcomes, like MBI and MAL, are heavily vulnerable to performance and detection bias. None of the 12 included RCTs achieved therapist blinding. While blinding therapists in physical and electrical stimulation trials presents technical challenges, the lack of therapist blinding introduces a major structural vulnerability to performance-related bias. This is particularly critical when evaluating activity-level outcomes, like MBI and MAL, where the unblinded factor may affect the results. Therapists aware of group allocations may unintentionally project differential expectations, deliver variable levels of participant encouragement during assessment, or inadvertently alter the quality of conventional therapy. Consequently, participant motivation and effort during activity performance tasks may be artificially heightened in active intervention groups. This performance-related bias and potential therapist-induced measurement expectations may have inflated the observed effects on activity-level outcomes. Therefore, the lack of therapist blinding across all included studies represents a methodological limitation that downgraded the certainty of evidence for activity-level outcomes in this review. Future RCTs should utilize therapist blinding when feasible or implement strict protocol standardization to minimize performance-related bias. Secondly, studies [40,45,46], lacking true intention-to-treat, might have overrated their results rather than reflecting the true effectiveness. Such risk of bias further downgrades the certainty of evidence of our findings.

**Strengths and limitations**

This study has several strengths. Firstly, qualitative and quantitative syntheses were conducted against different comparative conditions separately to provide a stratified and comprehensive comparison of the effects of the intervention compared with different comparative conditions. Secondly, outcomes were structured and reported according to the ICF domains, so that a comprehensive report on upper limb function recovery was established. Thirdly, the GRADE assessment for each meta-analysis would facilitate the decision-making in the design and implementation of the intervention in future studies and clinical practice.

Several limitations must be acknowledged. Firstly, our restriction to English-language publications introduces potential language bias. By excluding non-English publications, relevant evidence from non-English-speaking populations may have been omitted, potentially limiting the global generalizability of our findings. Secondly, the total number of included studies was small (*n* = 12), and pooled comparisons comprised far fewer than the recommended minimum threshold (10 studies) for reliable statistical tests for publication bias (such as funnel plot asymmetry tests or Egger’s regression tests) [63,64]. Consequently, small-study effects and potential publication bias, such as overrepresentation of small positive trials over unpublished null or negative findings, could not be ruled out and posed an inherent risk to the pooled estimates in this review. Future reviews and updates should encompass non-English studies and trial registries to capture emerging, unpublished, or ongoing evidence.

Thirdly, the persistent “moderate to high” heterogeneity in some of our meta-analyses downgraded the certainty of our findings. Since pooling the diverse dual-stimulation protocols risked masking subtle but meaningful parameter differences between studies, future RCTs should prioritize standardized stimulation protocols to allow for clearer cross-study and clinical translation.

In addition, the inclusion of broad stroke characteristics contributed to the heterogeneity and limited the precision of stroke characteristic-specific conclusions. The underlying neuroplastic recovery mechanisms and overall responsiveness to neuromodulation differ by stroke phases, such as early acute/subacute stroke involves rapid spontaneous neurological recovery and highly dynamic cortical reorganization, whereas chronic stroke relies on established compensatory mechanisms more [65]. Also, differences in baseline cortical state and neural network-level plasticity may result in varying effects on identical interventions [22,23,24,25]. As various stroke phases were included and several studies did not fully report baseline stroke characteristics (such as stroke type and affected sides), such differences in stroke characteristics across studies may affect the overall generalizability of our findings to specific clinical applicability and introduce a risk of clinical indirectness of our findings. Future RCTs should stratify cohorts by stroke characteristics and report them comprehensively.

Furthermore, despite the broad inclusion of different PES modalities being necessary to explore the overall landscape of combining central and peripheral electrical stimulation, pooling different PES modalities into a single PES umbrella category introduced a degree of heterogeneity into our findings. As the stimulation targets, neurological mechanisms, intensities, and therapeutic aims vary across modalities, the pooled estimates do not reflect a uniform clinical prescription and limit the immediate generalizability to specific clinical applications. While our subgroup analysis separating sensory-level and motor-level PES mitigated this limitation, certain levels of heterogeneity (I^2^ = 25% and I^2^ = 55%, respectively) remain due to variations in stimulation parameters. As more RCTs become available, future reviews should isolate and evaluate PES modalities individually to minimize heterogeneity and provide more precise therapeutic recommendations.

Nonetheless, the reliance on post-intervention-only pooling remains a methodological limitation. Post-intervention-only pooling was adopted in our quantitative synthesis because SD of changes between baseline and post-intervention were rarely reported by the included studies. Applying change scores or ANCOVA-adjusted effects requires wide-scale mathematical imputation of correlation coefficients, which may introduce significant calculation bias. Even though baseline comparability has been examined and no baseline imbalances were detected in all studies, unadjusted minor variations between groups may still be present and potentially reduce statistical precision, increase unexplained between-study variance, and limit the direct comparability across included studies in this review. Future RCTs should transparently report complete baseline, post-intervention, and change-score data to enable higher-precision meta-analyses.

Lastly, quantitative synthesis on some outcomes was heavily constrained due to limited studies (included only 2–5 studies) or incomplete data reported. For example, quantitative findings regarding spasticity reduction were fragile. Only two [42,43] out of five eligible studies reported sufficient data for quantitative synthesis on MAS. Quantitative findings on MAS were based on multiple arms from a single, with small sample size, study which were statistically fragile and limited its clinical generalizability. This limitation highlights the critical need for more robust and comprehensive data reporting in future RCTs.

## 5. Conclusions

In conclusion, this review revealed that current evidence, although of very low certainty, does not demonstrate a clear synergistic or additive effect of combined tDCS and PES on upper limb function recovery after stroke. Despite a very low certainty of evidence, current evidence suggests a potential enhancement of the therapeutic effect on ADL independence after stroke by combining tDCS and PES with standard rehabilitation therapy. However, given the low certainty of evidence, notable heterogeneity, and small number of included studies in the current evidence, future studies should employ factorial designs (consisting of at least the four arms of tDCS combined with PES, tDCS alone, PES alone, and sham or standard therapy control), better standardization and reporting of stimulation parameters, and individualized or state-dependent stimulation protocols to further investigate and verify the effects of combined tDCS and PES on upper limb function recovery after stroke.

## Figures and Tables

**Table 1:** Methodological quality of the included studies according to PEDro scale.

Study/Author (Year)	Item											Total Score	Overall Quality
	1	2	3	4	5	6	7	8	9	10	11		
Celnik 2009 [44]	Yes	Yes	No	Yes	Yes	No	Yes	Yes	Yes	Yes	Yes	8	Good
Halakoo 2021 [45]	Yes	Yes	No	Yes	Yes	No	Yes	Yes	No	Yes	Yes	7	Good
Liu 2025 [48]	Yes	Yes	No	Yes	No	No	No	Yes	Yes	Yes	Yes	6	Good
Menezes 2018 [38]	Yes	Yes	Yes	Yes	Yes	No	Yes	Yes	No	Yes	Yes	8	Good
Salazar 2020 [39]	Yes	Yes	Yes	Yes	Yes	No	Yes	Yes	Yes	Yes	Yes	9	Excellent
Sattler 2015 [40]	Yes	Yes	Yes	Yes	Yes	No	Yes	Yes	Yes	Yes	Yes	9	Excellent
Shaheiwola 2018 [46]	Yes	Yes	No	Yes	Yes	No	Yes	Yes	Yes	Yes	Yes	8	Good
Takebayashi 2017 [41]	Yes	Yes	Yes	Yes	No	No	Yes	Yes	No	Yes	Yes	7	Good
Wang 2025 [43]	Yes	Yes	Yes	Yes	No	No	Yes	Yes	Yes	Yes	Yes	8	Good
Wei 2022 [42]	Yes	Yes	Yes	Yes	Yes	No	Yes	Yes	Yes	Yes	Yes	9	Excellent
Yague 2023 [47]	Yes	Yes	No	Yes	Yes	No	Yes	Yes	No	Yes	Yes	7	Good
Zhao 2022 [49]	Yes	Yes	No	Yes	No	No	No	Yes	Yes	Yes	Yes	6	Good

Item 1: Eligibility criteria specified; Item 2: Subjects were randomly allocated to groups (in a crossover study, subjects were randomly allocated an order in which treatments were received); Item 3: Allocation was concealed; Item 4: the groups were similar at baseline regarding the most important prognostic indicators; Item 5: There was blinding of all subjects; Item 6: There was blinding of all therapists who administered the therapy; Item 7: There was blinding of all assessors who measured at least one key outcome; Item 8: Measures of at least one key outcome were obtained from more than 85% of the subjects initially allocated to groups; Item 9: All subjects for whom outcome measures were available received the treatment or control condition as allocated or, where this was not the case, data for at least one key outcome was analyzed by “intention to treat”; Item 10: The results of between-group statistical comparisons are reported for at least one key outcome; Item 11: The study provides both point measures and measures of variability for at least one key outcome.

**Table 2:** Characteristics of the included studies.

Author (Year)	Study Design	N Sex (M/F)	Interventions/Groups (n)	Outcomes	Key Findings
Celnik (2009) [44]	CRO	9 5M/4F	CN1: tDCS + PNS CN2: sham tDCS + PNS CN3: tDCS + sham PNS CN4: sham tDCS + sham PNS	FMST	CN1 led to improvement in accuracy in FMST than other conditions.
Halakoo (2021) [45]	P	35 ^a^ 21M/11F	EG1: a-tDCS + FES (12) EG2: sham tDCS + FES (11) CG: FES alone (12)	MAS, EMG	EG1 showed significant reduction in MAS at post-intervention and 1-month follow-up, compared to sham and control groups (*p* < 0.001). And EG1 showed an increase in EMG activities of FCR and ECR muscles during active wrist flexion and extension at post-intervention and 1-month follow-up when compared with sham and control groups (*p* < 0.001).
Liu (2025) [48]	P	90 33M/57F	EG1: b-tDCS + CCFES + CR (30) EG2: b-tDCS + CR (30) CG: CR alone (30)	FMA-WH, FTHUE-HK, MBI, Brunnstrom stages, Motor Assessment Scale, EMG	EG1 showed greater improvements in all outcomes than CR alone.
Menezes (2018) [38]	CRO	20 13M/7F	CN1: tDCS + RPSS > MT + FES CN2: sham + RPSS > MT + FES CN3: tDCS + sham > MT + FES CN4: sham + sham > MT + FES	Grasp strength, Pinch strength, ROM (wrist extension and flexion)	CN1 showed significant “time” effects on grasp strength of the paretic limb, and ROM (wrist extension) of the nonparetic limb at post-intervention.
Salazar (2020) [39]	P	30 20M/10F	EG: b-tDCS + FES (15) CG: sham tDCS + FES (15)	FMA-UE, Handgrip strength, MPM (MCT, RRV, PV), MQM (MS, TFI, ROM)	EG improved MCT (*p* = 0.039), mean reaching velocity (*p* = 0.022), grip strength (*p* = 0.034). Both experimental and control groups improved in mean returning velocity (*p* = 0.018), TFI (*p* = 0.022), MS (*p* = 0.001), and FMA-UE (*p* = 0.002).
Sattler (2015) [40]	P	20 14M/6F	EG: a-tDCS + RPNS (10) CG: sham tDCS + RPNS (10)	FMA-UE, FMA-WH, Grip strength, JTT, 9HPT, HTap, EMG	The time courses of motor recovery of the 2 groups were different and positively influenced (Group × Time interaction: *p* = 0.01). EG showed greater JTT improvement at 2-week and 4-week follow-up than CG.
Shaheiwola (2018) [46]	P	30 27M/3F	EG: b-tDCS + FES (15) CG: sham tDCS + FES (15)	cFMA, WMFT, MAS, EMG, TMS	EG demonstrated greater improvement in cFMA and WMFT to CG. No significant difference in spasticity (MAS) improvement between 2 groups (*p* = 0.081).
Takebayashi (2017) [41]	P	20 ^a^ 14M/6F	EG: b-tDCS + NMES + CIMT (10) CG: CIMT alone (9)	FMA-UE, MAL-AOU, MAL-QOM	b-tDCS + NMES enhanced CIMT effects on upper limb motor function, FMA-UE, (η2 = 0.43; *p* < 0.01), and MAL-AOU (η2 = 0.52; *p* = 0.02), but not significantly on MAL-QOM (η2 = 0.43; *p* = 0.07).
Wang (2025) [43]	P	90 56M/34F	EG1: tDCS + Acu-TENS-20 Hz (30) EG2: tDCS + Acu-TENS-100 Hz (30) CG: standard care (30)	MAS, MBI, FMA-UE&LE, EMG, CCR	EG showed a significant improvement in FMA-UE&LE at post-intervention (*p* < 0.001). Both EGs showed greater reductions in MAS than CG. EG2 showed a greater reduction in MAS than EG1. Both EGs showed an increase in MBI.
Wei (2022) [42]	P	26 18M/8F	EG1: b-tDCS + NMES + CR (9) EG2: b-tDCS + sham NMES + CR (9) CG: sham tDCS + sham NMES + CR (8)	FMA-UE, ARAT, MAS, MBI	EG1 showed significant within-group improvements on FMA-UE (*p* = 0.01) and ARAT (*p* = 0.03). No significant difference was shown on MAS and MBI across all groups.
Yague (2023) [47]	P	19 12M/7F	EG: b-tDCS + RPNS (10) CG: sham tDCS + RPNS (9)	FMA-UE, MAS, NIHSS, EMG, EEG	Both groups showed significant time changes in all outcomes (*p*< 0.05), but no significant differences between groups at any time point (*p* > 0.05).
Zhao (2022) [49]	P	60 39M/21F	EG: b-tDCS + FES + CR (30) CG: FES + CR (30)	FMA-WH, Brunnstrom stages	EG1 showed a significantly greater improvement in hemiplegic fingers’ functional evaluation (*p* = 0.026) and Brunnstrom stages (*p* < 0.001) than CG.

a-tDCS: anodal transcranial direct current stimulation; Acu-TENS: acupoints-based transcutaneous electrical nerve stimulation; ARAT: Action Research Arm Test; b-tDCS: bilateral transcranial direct current stimulation; CCR: co-contraction ratio; CCFES: contralaterally controlled functional electrical stimulation; cFMA: combined Fugl–Meyer Assessment-Upper Extremity (modified to exclude the coordination, speed, and reflexes scores); CG: control group; CIMT: Constraint-Induced Movement Therapy; CN: condition; CR: conventional rehabilitation; CRO: cross-over; d: day(s); ECR: extensor carpi radialis; EEG: electroencephalogram; EG: experimental group; EMG: electromyography; F: female; FCR: flexor carpi radialis; FES: functional electrical stimulation; FMST: finger motor sequence task; FMA-UE: Fugl–Meyer Assessment-Upper Extremity; FMA-UE&LE: Fugl–Meyer Assessment-Upper Extremity and Lower Extremity; FMA-WH: Fugl–Meyer Assessment (wrist and hand parts); FTHUE-HK: Functional Test for the Hemiplegic Upper Extremity; HM: hemorrhagic stroke; Htap: Hand tapping test; IQR: interquartile range; IS: ischemic stroke; JTT: Jebsen–Taylor Hand Function Test; L: left; M: male; MAL-AOU: motor activity log—amount of use scale; MAL-QOM: motor activity log—quality of movement; MAS: Modified Ashworth Scale; MBI: Modified Barthel Index; MCT: movement cycle time; MPM: motor performance measures; MQM: movement quality measures; MS: movement smoothness; MT: motor training; m: month(s); NIHSS: National Institutes of Health Stroke Scale; NMES: neuromuscular electrical stimulation; NR: not reported; P: parallel; PNS: peripheral nerve stimulation; PV: peak velocity; R: right; ROM: range of movement; RPNS: repetitive peripheral nerve stimulation; RPSS: repetitive peripheral nerve sensory stimulation; RRV: reaching and returning velocity; SD: standard deviation; tDCS: transcranial direct current stimulation; TFI: trunk forward inclination; WMFT: Wolf Motor Function Test; y: year(s); 9HPT: 9 Hole Peg Test. ^a^ Initial sample size before attrition, data after attrition were not reported and analyzed according to the intention-to-treat principle.

**Table 3:** Intervention parameters and results of the included studies.

Author (Year)	Type of tDCS	tDCS Parameters	Type of PES	PES Parameters	Sessions	Side Effect	Results
Celnik (2009) [44]	a-tDCS	Device: Iomed Phoresor PM850 Site: AN (ipsilesional M1)/CT (contralateral supraorbital area) Electrode size: 3”× 3” Intensity: 1mA Duration: 20 mins	PNS	Device: Grass stimulator S8800 (Astro-Med Inc.) Site: Median and ulnar nerve (paretic side) Electrode size: NR Frequency: 10 Hz Pulse width: 1 ms Duty cycle: 1:1 Intensity: To elicit small CMAP (50–100 µV) in APB and FDI without visible muscle twitches. Duration: 120 mins	4 (6.3 ± 0.9 days apart)	No	tDCS + PNS led to improvement in accuracy in FMST than other conditions.
Halakoo (2021) [45]	a-tDCS	Device: NR Site: AN (ipsilesional M1)/CT (contralateral supraorbital area) Electrode size: 5 × 7 cm Intensity: 2 mA Duration: 20 mins	FES	Device: NR Site: ECR muscle (paretic side) Electrode size: 4 × 6 cm Frequency: 50 Hz Pulse width: 250 µs Duty cycle: 6 s on: 12 s off Intensity: NR Duration: 20 mins	10 (5 d/w, for 2 w)	Tingling, itching	tDCS + FES showed significant reduction in MAS and EMG activities of FCR with wrist resting position, at post-intervention and 1-month follow-up, compared to sham and control groups (*p* < 0.001). And tDCS + FES showed an increase in EMG activities of FCR and ECR muscles during active wrist flexion and extension increased at post-intervention and 1-month follow-up when compared with sham and control groups (*p* < 0.001).
Liu (2025) [48]	b-tDCS	Device: Model DK-501 (Shijiazhuang Dukon Medical Instrument Co., Ltd.) Site: AN (ipsilesional hand area)/CT (contralateral hand area) Electrode size; 5 × 7 cm Intensity: 1.5–2 mA Duration: 20 mins	CCFES	Device: Model S4-30 (Nanjing Weisi Medical Technology Co., Ltd.) Site: Motor point of wrist extensor dorsi muscles (both sides) Frequency: 60 Hz Pulse width: 200 µs Duty cycle: 10 s on: 10 s off Intensity: At a level to produce an extension amplitude equivalent to the unaffected side Duration: 20 mins	7–14 (1/day, for 7–14 days)	NR	tDCS + CCFES + CR showed greater improvements in all outcomes than CR alone.
Menezes (2018) [38]	a-tDCS	Device: Neurocom tDCS DC-Stimulator Site: AN (ipsilesional M1)/CT (contralateral supraorbital area) Electrode size: NR Intensity: 1 mA Duration: 20 mins	RPSS	Device: Square Pulse Stimulator (Grass Instrument Division of Astro-med Inc.) Site: Median, ulnar, radial nerves (paretic side) Electrode size: NR Frequency: 10 Hz Pulse width: 1 ms Duty cycle: 1:1 Intensity: Paresthesia induced without visible muscle movement and pain Duration: 120 mins	4 (10–15 days separated)	NR	tDCS + RPSS showed significant “time” effects on grasp strength of the paretic limb, and ROM (wrist extension) of the nonparetic limb at post-intervention.
			FES	Device: FESMED IV, Brazil Site: EDC Electrode size: 3 × 5 cm Frequency: 40 Hz Pulse width: 250 µs Duty cycle: 1 s on: 1.5 s off Intensity: NR Duration: 25 mins			
Salazar (2020) [39]	b-tDCS	Device: TCT neurostimulator (Research Version), TransCranial Research Ltd. Site: AN (ipsilesional M1)/CT (contralesional M1) Electrode size: 5 × 5 cm Intensity: 2 mA Duration: 30 mins	FES	Device: Dualpex-071, Quark Medical, Brazil Site: Anterior deltoid, serratus anterior, triceps brachii, wrist extensor muscles (paretic side) Frequency: 40 Hz Pulse width: 300 µs Duty cycle: 1:2 Intensity: maximum tolerated Duration: 30 mins	10 (5/week, for 2 weeks)	Tingling during tDCS (mild to moderate)	tDCS + FES improved MCT (*p* = 0.039), mean reaching velocity (*p* = 0.022), grip strength (*p* = 0.034). Both experimental and control groups improved in mean returning velocity (*p* = 0.018), TFI (*p* = 0.022), MS (*p* = 0.001), and FMA-UE (*p* = 0.002).
Sattler (2015) [40]	a-tDCS	Device: DC stimulator plus, Magstim Site: AN (ECR hotspot on the ipsilesional M1)/CT (contralesional supraorbital area) Electrode size: 35 cm^2^ Intensity: 1.2 mA Duration: 13 mins	RPNS	Device: DIGITIMER DS7A Site: Radial nerve (paretic side) Electrode size: 2 cm (diameter) Frequency: 5 Hz Pulse width: NR Duty cycle: NR Intensity: <0.7 x MT Duration: 13 mins	5 (5 consecutive days)	Tingling	The time courses of motor recovery of the 2 groups were different and positively influenced (Group × Time interaction: *p* = 0.01). EG showed greater JTT improvement at 2-week and 4-week follow-up than CG.
Shaheiwola (2018) [46]	b-tDCS	Device: BrainStim stimulator, EMS s.r.l., Italy Site: AN (APB hotspot on the ipsilesional M1)/CT (symmetrical area on the contralesional M1) Electrode size: 5 × 5 cm Intensity: 2 mA Duration: 20 mins	FES	Device: MyoNet-BOW-III, NCC Medical Co., Ltd., China Site: Multiple sites along the paretic upper limb Electrode size: NR Frequency: 40 Hz Pulse width: 250 µs Duty cycle: NR Intensity: Adjusted weekly based on clinical needs and motor levels Duration: 60 mins	20 (5 d/w, for 4 w)	No	EG demonstrated greater improvement in cFMA and WMFT to CG. No significant difference in spasticity (MAS) improvement between 2 groups (*p* = 0.081).
Takebayashi (2017) [41]	b-tDCS	Device: DC-STIMULATOR PLUS (neuroConn GmbH, Ilmenau, Germany) Site: AN (C3/C4 on ipsilesional M1)/CT (C3/C4 on contralesional M1) Electrode size: NR Intensity: 1 mA (constant) Duration: 20 mins	NMES	Device: TORIO stimulation system (Ito Co., Ltd., Tokyo, Japan) Site: EDM (paretic side) Electrode size: NR Frequency: 20 Hz Pulse width: 300 µs Duty cycle: 1:1 Intensity: At a level of minimal visible muscle contraction and mild paresthesia, without pain Duration: 10 mins	20 (2/d, 5 d/w, for 2 w)	No	b-tDCS + NMES enhanced CIMT effects on upper limb motor function, FMA-UE, (η2 = 0.43; *p* < 0.01), and MAL-AOU (η2 = 0.52; *p* = 0.02), but not significantly on MAL-QOM (η2 = 0.43; *p* = 0.07).
Wang (2025) [43]	a-tDCS	Device: NeuStim NSS10E, Neuracle Tech (Changzhou) Co., Ltd., Changzhou, China Site: AN (upper 3/5 of MS6 on ipsilesional hemisphere)/CT (contralateral supraorbital area) Electrode size: AN (5 × 10 cm)/CT (5 × 5 cm) Intensity: 1 mA Duration: 20 mins	Acu-TENS	Device: QL/T-IIA, Sichuan Qianli Beoka Medical Tech. Co., Ltd., Chengdu, China Site: Acupoints (LI11, TE5, GB34, ST37) on the paretic side Electrode size: 4 × 4 cm Frequency: EG1(20 Hz)/EG2(100 Hz) Pulse width: 0.2 ms Duty cycle: NR Intensity: maximum tolerated Duration: 30 mins	12 (3 d/w, for 4 w)	No	EG showed a significant improvement in FMA-UE&LE at post-intervention (*p* < 0.001). Both EGs showed greater reductions in MAS than CG. EG2 showed a greater reduction in MAS than EG1. Both EGs showed an increase in MBI.
Wei (2022) [42]	b-tDCS	Device: Intelect Mobile Stimulation and Combination, DJO, France Site: AN (ipsilesional M1); CT (contralesional M1) Electrode size: 25 cm^2^ Intensity: 2 mA Duration: 30 mins	NMES	Device: Endomed-182; Enraf Nonius, Netherlands Site: Motor point of ECRB and EDM (paretic side) Electrode size: 2 cm (diameter) Frequency: 50 Hz Pulse width: 200 μs Duty cycle: 10 s on: 10 s off Intensity: 10–20 mA Duration: 30 mins	15 (5 d/w, for 3 w)	No	EG1 showed significant within-group improvements on FMA-UE (*p* = 0.01) and ARAT (*p* = 0.03). No significant difference was shown on MAS and MBI across all groups.
Yague (2023) [47]	b-tDCS	Device: neuroConn DCStimulator Site: AN (C3/C4 on ipsilesional M1)/CT (contralesional M1) Electrode size: 25 cm^2^ Intensity: 2 mA Duration: 20 mins	RPNS	Device: INNERVATOR 252 Site: Radial nerve (paretic side) Electrode size: 5 cm diameter Frequency: 100 Hz Pulse width: 1 ms Duty cycle: 0.4 s on: 6 s off Intensity: 20–30 mA (to elicit visible wrist extension) Duration: 20 mins	5 (5 consecutive days)	No	Both groups showed significant time-changes in all outcomes (*p* < 0.05), but no significant differences between groups at any time point (*p* > 0.05).
Zhao (2022) [49]	b-tDCS	Device: Jiangxi Huaheng Jingxing Med. Tech. Co., Ltd., China Site: AN (hand area of ipsilesional M1)/CT (hand area of contralesional M1) Electrode size: NR Intensity: 2 mA Duration: 20 mins	FES	Device: smart joint training device (Beijing Zhongjian Gaemi Tech. Co., Ltd., China) Site: Wrist’s dorsal muscles (paretic side) Electrode size: NR Frequency: NR Pulse width: NR Duty cycle: NR Intensity: Based on patient’s tolerance Duration: 20 mins	12 (6 d/w, 2 w)	Tingling (slight)	EG1 showed a significantly greater improvement in hemiplegic fingers’ functional evaluation (*p* = 0.026) and Brunnstrom stages (*p* < 0.001) than CG.

Acu-TENS: acupoints-based transcutaneous electrical nerve stimulation; AN: anode; APB: Abductor pollicis brevis; ARAT: Action Research Arm Test; a-tDCS: anodal transcranial direct current stimulation; b-tDCS: bilateral transcranial direct current stimulation; CCFES: contralateral controlled functional electrical stimulation; cFMA: combined Fugl–Meyer Assessment-Upper Extremity (modified to exclude the coordination, speed, and reflexes scores); CG: control group; CIMT: Constraint-Induced Movement Therapy; CMAPs: compound muscle action potential; CN: Condition; CR: conventional rehabilitation; d: day(s); ECR: extensor carpi radialis; ECRB: extensor carpi radialis brevis; EDC: extensor digitorum communis; EDM: extensor digitorum muscle; EG: experimental group; EMG: electromyography; FCR: flexor carpi radialis; FDI: First dorsal interosseus; FES: functional electrical stimulation; FMST: finger motor sequence task; FMA-UE: Fugl–Meyer Assessment-Upper Extremity; FMA-UE&LE: Fugl–Meyer Assessment-Upper Extremity and Lower Extremity; JTT: Jebsen–Taylor Hand Function Test; M1: Primary motor cortex; MAL-AOU: motor activity log—amount of use scale; MAL-QOM: motor activity log—quality of movement; MAS: Modified Ashworth Scale; MBI: Modified Barthel Index; MCT: movement cycle time; MS: movement smoothness; MS6: the anterior oblique line of the vertex-temporal; MT: motor threshold; NMES: neuromuscular electrical stimulation; NR: not reported; PNS: peripheral nerve stimulation; ROM: range of movement; RPNS: repetitive peripheral nerve stimulation; RPSS: repetitive peripheral nerve sensory stimulation; TFI: trunk forward inclination; tDCS: transcranial direct current stimulation; w: week(s); WMFT: Wolf Motor Function Test.

**Table 4:** GRADE evidence profile of tDCS + motor PES versus tDCS alone.

No. of Studies	tDCS + PES N	Control N	SMD/MD (95% CI)	Risk of Bias	Inconsistency	Indirectness	Imprecision	Other Considerations	Certainty of Evidence
**Exploratory analysis: FMA (FMA-UE and FMA-WH)**									
2	39	39	0.19 (−6.71 to 7.09)	Serious ^a^	Serious ^b^	Not serious	Serious ^c^	none	⨁◯◯◯ Very low ^a,b,c^
**ADL (MBI)**									
2	39	39	8.30 (−18.04 to 34.63)	Serious ^a^	Not serious	Not serious	Serious ^c^	none	⨁⨁◯◯ Low ^a,c^

N: sample size; CI: confidence interval; SMD: standardized mean difference; MD: mean difference; ^a^: downgraded one level due to serious risk of bias; ^b^: downgraded one level due to serious inconsistency; ^c^: downgraded one level due to serious imprecision; ⨁: indicating present certainty; ◯: indicating downgraded/absent certainty.

**Table 5:** GRADE evidence profile of tDCS + motor/sensory PES versus PES alone.

No. of Studies	tDCS + PES N	Control N	SMD/MD (95% CI)	Risk of Bias	Inconsistency	Indirectness	Imprecision	Other Considerations	Certainty of Evidence
**Exploratory analysis: FMA (FMA-UE and FMA-WH)**									
5	79	79	0.02 (−0.57 to 0.61)	serious ^a^	Not serious	Not serious	serious ^c^	none	⨁⨁◯◯ Low ^a,c^
**Exploratory subgroup analysis: FMA-UE**									
3	39	39	−0.61 (−12.38 to 11.16)	Serious ^a^	Not serious	Not serious	Serious ^c^	none	⨁⨁◯◯ Low ^a,c^
**Exploratory subgroup analysis: FMA-WH**									
2	40	40	0.68 (−26.25 to 27.61)	Serious ^a^	Serious ^b^	Not serious	Serious ^c^	none	⨁◯◯◯ Very low ^a,b,c^
**Exploratory subgroup analysis: FMA (Chronic stroke)**									
2	30	30	−0.11 (−5.01 to 4.80)	Serious ^a^	Not serious	Not serious	Serious ^c^	none	⨁⨁◯◯ Low ^a,c^
**Exploratory subgroup analysis: FMA (Acute/Subacute stroke)**									
3	49	49	0.09 (−1.23 to 1.42)	Serious ^a^	Serious ^b^	Not serious	Serious ^c^	none	⨁◯◯◯ Very low ^a,b,c^

N: sample size; CI: confidence interval; SMD: standardized mean difference; MD: mean difference; ^a^: downgraded one level due to serious risk of bias; ^b^: downgraded one level due to serious inconsistency; ^c^: downgraded one level due to serious imprecision; ⨁: indicating present certainty; ◯: indicating downgraded/absent certainty.

**Table 6:** GRADE evidence profile of tDCS + motor PES versus PES alone.

No. of Studies	tDCS + PES N	Control N	SMD/MD (95% CI)	Risk of Bias	Inconsistency	Indirectness	Imprecision	Other Considerations	Certainty of Evidence
**FMA-UE**									
2	30	30	−1.09 (−50.61 to 48.43)	Serious ^a^	Not serious	Not serious	Serious ^c^	none	⨁⨁◯◯ Low ^a,c^
**Exploratory analysis: FMA (FMA-UE and FMA-WH)**									
3	60	60	0.12 (−1.12 to 1.35)	Serious ^a^	Serious ^b^	Not serious	Serious ^c^	none	⨁◯◯◯ Very low ^a,b,c^

N: sample size; CI: confidence interval; SMD: standardized mean difference; MD: mean difference; ^a^: downgraded one level due to serious risk of bias; ^b^: downgraded one level due to serious inconsistency; ^c^: downgraded one level due to serious imprecision; ⨁: indicating present certainty; ◯: indicating downgraded/absent certainty.

**Table 7:** GRADE evidence profile of tDCS + sensory PES versus PES alone.

No. of Studies	tDCS + PES N	Control N	SMD/MD (95% CI)	Risk of Bias	Inconsistency	Indirectness	Imprecision	Other Considerations	Certainty of Evidence
**Exploratory analysis: FMA (FMA-UE and FMA-WH)**									
2	19	19	−0.21 (−5.04 to 4.61)	Serious ^a^	Not serious	Not serious	Not serious	none	⨁⨁⨁◯ Moderate ^a^

N: sample size; CI: confidence interval; SMD: standardized mean difference; MD: mean difference; ^a^: downgraded one level due to serious risk of bias; ⨁: indicating present certainty; ◯: indicating downgraded/absent certainty.

**Table 8:** GRADE evidence profile of tDCS + PES versus standard therapy alone.

No. of Studies	tDCS + PES N	Control N	SMD/MD (95% CI)	Risk of Bias	Inconsistency	Indirectness	Imprecision	Other Considerations	Certainty of Evidence
**Exploratory analysis: tDCS + motor/sensory PES vs. standard therapy alone (ADL)**									
2	90	60	14.37 (1.83 to 26.91) *	Serious ^a^	Serious ^b^	Not serious	Serious ^c^	none	⨁◯◯◯ Very low ^a,b,c^
**Exploratory analysis: tDCS + sensory PES vs. standard therapy alone (FMA-UE&LE)**									
2 comparisons from 1 study	60	30	10.57 (−32.29 to 53.43)	Serious ^a^	Not serious	Not serious	Serious ^c^	none	⨁⨁◯◯ Low ^a,c^
**Exploratory analysis: tDCS + sensory PES vs. standard therapy alone (MAS)**									
2 comparisons from 1 study	60	30	−2.29 (−13.09 to 8.51)	Serious ^a^	Not serious	Not serious	Serious ^c^	none	⨁⨁◯◯ Low ^a,c^
**Exploratory analysis: tDCS + sensory PES vs. standard therapy alone (ADL)**									
2 comparisons from 1 study	60	30	12.16 (−44.69 to 69.02)	Serious ^a^	Not serious	Not serious	Serious ^c^	none	⨁⨁◯◯ Low ^a,c^

* *p* < 0.05; N: sample size; CI: confidence interval; SMD: standardized mean difference; MD: mean difference; ^a^: downgraded one level due to serious risk of bias; ^b^: downgraded one level due to serious inconsistency; ^c^: downgraded one level due to serious imprecision; ⨁: indicating present certainty; ◯: indicating downgraded/absent certainty.

**Table 9:** Results of sensitivity analyses using the “leave-one-out” method.

SMD/MD; (95% CI); *p*; I^2^		Difference	Excluded Study/Arm
Primary Meta-Analysis Results	Sensitivity Analysis Results		
**tDCS+ motor PES vs. PES alone (FMA)**			
0.12; (−1.12 to 1.35); 0.72; 55%	0.40; (−0.68 to 1.47); 0.13; 0%	Similar effect that remains non-significant; Reduced heterogeneity	Salazar 2020 [39]
	0.02; (−6.00 to 6.04); 0.98; 77%	Similar effect that remains non-significant; Increased heterogeneity	Shaheiwola 2018 [46]
	−0.11; (−5.01 to 4.80); 0.83; 54%	Opposite effect that remains non-significant; Similar heterogeneity	Zhao 2022 [49]
**tDCS+ motor/sensory PES vs. PES alone (FMA)**			
0.02; (−0.57 to 0.61); 0.93; 42%	0.18; (−0.50 to 0.86); 0.46; 24%	Similar effect that remains non-significant; Reduced heterogeneity	Salazar 2020 [39]
	0.14; (−0.54 to 0.82); 0.56; 33%	Similar effect that remains non-significant; Reduced heterogeneity	Sattler 2015 [40]
	−0.06; (−0.90 to 0.77); 0.83; 54%	Opposite effect that remains non-significant; Increased heterogeneity	Shaheiwola 2018 [46]
	−0.02; (−0.87 to 0.82); 0.94; 56%	Opposite effect that remains non-significant; Increased heterogeneity	Yague 2023 [47]
	−0.15; (−0.86 to 0.56); 0.55; 16%	Opposite effect that remains non-significant; Reduced heterogeneity	Zhao 2022 [49]
**tDCS+ motor/sensory PES vs. standard therapy alone (ADL)**			
14.37; (1.83 to 26.91); 0.039 *; 71%	12.16; (−44.69 to 69.02); 0.224; 69%	Similar effect that becomes non-significant; Similar heterogeneity	Liu 2025 [48]
	17.05; (16.08 to 18.03); 0.003 *; 0%	Similar effect that remains significant; Reduced heterogeneity	Wang 2025 (20 Hz PES) [43]
	13.01: (−45.71 to 71.73); 0.217; 86%	Similar effect that becomes non-significant; Increased heterogeneity	Wang 2025 (100 Hz PES) [43]

* *p* < 0.05; SMD: standardized mean difference; MD: mean difference; CI: confidence interval; *p*: *p*-value of the effect size; I^2^: heterogeneity; Wang 2025 (20 Hz PES): the comparison of “tDCS + Acu-TENS (20 Hz)” vs. “standard care” in Wang (2025) [43]; Wang 2025 (100 Hz PES): the comparison of “tDCS + Acu-TENS (100 Hz)” vs. “standard care” in Wang (2025) [43].

**Table 10:** Sensitivity analyses using the standard DerSimonian–Laird (DL) random-effects model via RevMan.

No. of Pooled Studies	SMD/MD; (95% CI); *p*; I^2^		Difference
	REML + HK Method by R	DL Method by RevMan	
**tDCS+ motor PES vs. tDCS alone (FMA)**			
2	0.19; (−6.71 to 7.09); 0.79; 75%	0.19; (−0.88 to 1.25); 0.73; 75%	Effect size (SMD) remains non-significant
**tDCS+ motor PES vs. tDCS alone (ADL)**			
2	8.30; (−18.04 to 34.63); 0.16; 18%	8.30; (4.23 to 12.36); <0.001 *; 18%	Non-significant effect size (MD) becomes significant
**tDCS+ motor PES vs. PES alone (FMA-UE)**			
2	−1.09; (−50.61 to 48.43); 0.83; 54%	−1.09; (−8.73 to 6.55); 0.78; 54%	Effect size (MD) remains non-significant
**tDCS+ motor PES vs. PES alone (FMA)**			
3	0.12; (−1.12 to 1.35); 0.72; 55%	0.12; (−0.44 to 0.68); 0.68; 55%	Effect size (SMD) remains non-significant
**tDCS+ sensory PES vs. PES alone (FMA)**			
2	−0.21; (−5.04 to 4.61); 0.67; 25%	−0.21; (−0.96 to 0.53); 0.57; 25%	Effect size (SMD) remains non-significant
**tDCS+ motor/sensory PES vs. PES alone (FMA)**			
5	0.02; (−0.57 to 0.61); 0.93; 42%	0.02; (−0.41 to 0.45); 0.92; 42%	Effect size (SMD) remains non-significant
**tDCS+ motor/sensory PES vs. standard therapy alone (ADL)**			
2	14.37; (1.83 to 26.91); 0.039 *; 71%	14.39; (8.91 to 19.88); <0.001 *; 71%	Effect size (MD) remains significant
**tDCS+ sensory PES vs. standard therapy alone (MAS)**			
2 comparisons from 1 single study	−2.29; (−13.09 to 8.51); 0.23; 57%	−2.29; (−3.95 to −0.62); 0.007 *; 57%	Non-significant effect size (MD) becomes significant
**tDCS+ sensory PES vs. standard therapy alone (ADL)**			
2 comparisons from 1 single study	12.16; (−44.69 to 69.02); 0.224; 69%	12.16; (3.39 to 20.93); 0.007 *; 69%	Non-significant effect size (MD) becomes significant

* *p* < 0.05; SMD: standardized mean difference; MD: mean difference; CI: confidence interval; *p*: *p*-value of the effect size; I^2^: heterogeneity.

**Table 11:** Proposed minimum reporting standard for combined tDCS and PES for stroke rehabilitation.

Categories	Recommended Parameters to Be Reported	Description of Minimum Reporting Standard
tDCS parameters	Current intensity and density	Current (mA), electrode area (cm^2^), and current density (mA/cm^2^) to determine local charge delivery.
	Electrode montage and sites	Positioning for the anode and cathode.
	Duration	Duration per session.
PES parameters	PES modality	Report on the exact PES categorization (e.g., FES, NMES, PNS, RPNS, RPSS, TENS, etc.).
	Waveform parameters and intensity	Pulse frequency (Hz), pulse width, and duty cycle.
	Electrode sizes and sites	Electrode size (cm^2^), electrode placement (e.g., targeted muscle groups or peripheral nerves with anatomical landmarks reported).
	Intensity threshold	To clarify the sensory-level (e.g., paresthesia without muscle contraction) or motor-level (e.g., evoked muscle twitch/contraction) stimulation.
	Duration	Duration per session.
Stimulation protocol	Timing synchronization	Report on the delivery timing of stimulations (e.g., simultaneously or sequentially).
	Concurrent rehabilitation	Protocol, content, and duration of concurrent rehabilitation therapy (if any) must be reported. The timing synchronization with stimulations must be reported too.
	Overall duration and dosage	Total no. of sessions, frequency (sessions/week), and complete duration (weeks).
Participant’s stroke characteristics	Stroke type	Stroke type must be reported (ischemic or hemorrhagic).
	Stroke chronicity	Stroke phase (acute, subacute, chronic), post-stroke time.
	Lesion characteristics and affected side	Lesion location (thus, affected side) must be reported.

## Data Availability

All relevant data are shown in this article and the Supplementary Materials.

## Supplementary Materials

The following supporting information can be downloaded at: https://www.mdpi.com/article/10.3390/brainsci16080791/s1, Table S1: PRISMA 2020 Checklist; Table S2: Details of search strategy; Table S3: Detailed characteristics of included studies.

## Abbreviations

The following abbreviations are used in this manuscript:

ADL	Activities of Daily Living
AOU	Amount of Use
ARAT	Action Research Arm Test
BI	Barthel Index
CI	Confidence Interval
CR	Conventional Rehabilitation
ES	Electrical Stimulation
FES	Functional Electrical Stimulation
FMA	Fugl–Meyer Assessment
FMA-UE	Fugl–Meyer Assessment—Upper Extremity
FMA-WH	Fugl–Meyer Assessment—Wrist/Hand
FMA-UE&LE	Fugl–Meyer Assessment—Upper Extremity and Lower Extremity
FMST	Finger Motor Sequence Task
GRADE	Grading of Recommendations Assessment, Development and Evaluation
ICF	International Classification of Functioning, Disability and Health
MAS	Modified Ashworth Scale
MBI	Modified Barthel Index
MD	Mean Difference
MAL	Motor Activity Log
MQ	Methodological Quality
NMES	Neuromuscular Electrical Stimulation
PEDro	Physiotherapy Evidence Database
PES	Peripheral Electrical Stimulation
PICOS	Population, Intervention, Comparison, Outcome, Study design
PNS	Peripheral Nerve Stimulation
PRISMA	Preferred Reporting Items for Systematic Reviews and Meta-Analyses
QOM	Quality of Movement
RCT	Randomized Controlled Trial
ROB	Risk of Bias
RoB 2	Cochrane Risk-of-Bias Tool for Randomized Trials, Version 2
RPNS	Repetitive Peripheral Nerve Stimulation
RPSS	Repetitive Peripheral Nerve Sensory Stimulation
SD	Standard Deviation
SMD	Standardized Mean Difference
tDCS	Transcranial Direct Current Stimulation
WMFT	Wolf Motor Function Test
