# Supplementary material for: Effects of Transcranial Direct Current Stimulation Combined with Peripheral Electrical Stimulation on Upper Limb Function Recovery in Stroke: A Systematic Review and Meta-Analysis
Source: Brain Sci. 2026 Jul 27;16(8):791. doi: 10.3390/brainsci16080791 (PMC13511701; doi:10.3390/brainsci16080791)
Supplement: Supplementary file 1 [file brainsci-16-00791-s001.zip › Table S3.pdf]

Supplementary Material Table S3

Table S3. Characteristics of the included studies.

Author (Year)	Study design	N Sex (M/F)	Age (years) Mean ± SD	Stroke characteristics Phase Duration [Mean±SD/median (IQR)] Type (IS/HM) Affected side (L/R)	Interventions / Groups (n)	Outcomes	Assessment period
Celnik (2009) [44]	CRO	9<br>5M/4F	55.3 ± 3.76	Chronic<br>55.7±5.57 (m)<br>9IS / 0HM<br>4L / 5R	CN1: tDCS + PNS<br>CN2: sham tDCS + PNS<br>CN3: tDCS + sham PNS<br>CN4: sham tDCS + sham PNS	FMST	Pre, 30 mins post, 24 hours post, 6.3±0.5 days post
Halakoo (2021) [45]	P	35[a]<br>21M/11F	EG1: 61.83 ± 7.60<br>EG2: 58.80 ± 5.23<br>CG: 67.20 ± 4.96	Subacute<br>NR<br>NR<br>NR	EG1: a-tDCS + FES (12)<br>EG2: sham tDCS + FES (11)<br>CG: FES alone (12)	MAS, EMG	Pre, post, 1-month follow-up
Liu (2025) [48]	P	90<br>33M/57F	EG1: 59.43±10.52<br>EG2: 55.53±11.09<br>CG: 58.53±11.27	Subacute<br>EG1: 6.63±2.63;<br>EG2: 5.87±2.61; CG: 7.30±2.67 (d)<br>NR<br>NR	EG1: b-tDCS + CCFES + CR (30)<br>EG2: b-tDCS + CR (30)<br>CG: CR alone (30)	FMA-WH, FTHUE-HK, MBI, Brunnstrom stages, Motor Assessment Scale, EMG	Pre, post, 1-month follow-up, 3-month follow-up, 6-month follow-up
Menezes (2018) [38]	CRO	20<br>13M/7F	56.6 ± 12.3	Chronic<br>5.7±5.7 (y)<br>16IS / 4HM<br>8L / 12R	CN1: tDCS + RPSS > MT + FES<br>CN2: sham + RPSS > MT + FES<br>CN3: tDCS + sham > MT + FES<br>CN4: sham + sham > MT + FES	Grasp strength, Pinch strength, ROM (wrist extension and flexion)	Pre, post
Salazar (2020) [39]	P	30<br>20M/10F	EG: 60 ± 10.34<br>CG: 56 ± 16.08	Chronic<br>EG: 21(6-59); CG: 23(8-59) (m)<br>25IS / 5HM<br>16L / 14R	EG: b-tDCS + FES (15)<br>CG: sham tDCS + FES (15)	FMA-UE, Handgrip strength, MPM (MCT, RRV, PV), MQM (MS, TFI, ROM)	Pre, post
Sattler (2015) [40]	P	20<br>14M/6F	EG: 67.6 ± 10<br>CG: 62.7 ± 12	Acute<br>EG: 5.3±2.6; CG: 5.6±3.8 (d)<br>NR<br>NR	EG: a-tDCS + RPNS (10)<br>CG: sham tDCS + RPNS (10)	FMA-UE, FMA-WH, Grip strength, JTT, 9HPT, HTap, EMG	Pre, post, 2-week follow-up, 4-week follow-up
Shaheiwola (2018) [46]	P	30<br>27M/3F	EG: 49.3 ± 9.4<br>CG: 51.9 ± 11	Chronic<br>EG: 18(15); CG: 16(13) (m)<br>NR<br>14L / 16R	EG: b-tDCS + FES (15)<br>CG: sham tDCS + FES (15)	cFMA, WMFT, MAS, EMG, TMS	Pre, post
Takebayashi (2017) [41]	P	20[a]<br>14M/6F	EG: 58.90 ± 8.28<br>CG: 59.7 ± 15.82	Chronic<br>EG: 922.30±693.95; CG: 1195.7 ±1546.48 (d)<br>14IS / 6HM<br>10L / 10R	EG: b-tDCS + NMES + CIMT (10)<br>CG: CIMT alone (9)	FMA-UE, MAL-AOU, MAL-QOM	Pre, post
Wang (2025) [43]	P	90<br>56M/34F	EG1: 62.8 ± 11.52<br>EG2: 63.6 ± 15.22<br>CG: 69.57 ± 16.09	Subacute / Chronic<br>EG1: 5(2-9); EG2: 5(2-8.25);<br>CG:6 (2-9.25) (m)<br>90IS / 0HM<br>47L / 43R	EG1: tDCS + Acu-TENS-20Hz (30)<br>EG2: tDCS + Acu-TENS-100Hz (30)<br>CG: standard care (30)	MAS, MBI, FMA-UE&LE, EMG, CCR	Pre, post, 4-week follow-up
Wei (2022) [42]	P	26<br>18M/8F	EG1: 63.9 ± 9<br>EG2: 54.4 ± 16.6<br>CG: 68.4 ± 7.5	Chronic<br>EG1: 19.2(23.9);<br>EG2: 25.9(32.4);<br>CG: 17.8(20.2) (m)<br>26IS / 0HM<br>14L / 12R	EG1: b-tDCS + NMES + CR (9)<br>EG2: b-tDCS + sham NMES + CR (9)<br>CG: sham tDCS + sham NMES + CR (8)	FMA-UE, ARAT, MAS, MBI	Pre, post, 1-month follow-up
Yague (2023) [47]	P	19<br>12M/7F	EG: 56 ± 14.2<br>CG: 59.4 ± 12.6	Acute / Subacute<br>EG: 10±4.9; CG: 9.3±3.2 (d)<br>10IS / 9HM	EG: b-tDCS + RPNS (10)<br>CG: sham tDCS + RPNS (9)	FMA-UE, MAS, NIHSS, EMG, EEG	Pre, post, 3-month follow-up, 6-month

				NR			follow-up, 12-month follow-up
Zhao (2022) [49]	P	60<br>39M/21F	EG: 59.57 ± 10.41<br>CG: 62.87 ± 8.4	Acute / Subacute<br>EG: 7.67±2.64; CG: 7.57±2.75 (d)<br>60IS / 0HM<br>34L / 26R	EG: b-tDCS + FES + CR (30)<br>CG: FES + CR (30)	FMA-WH, Brunnstrom stages	Pre, post

a-tDCS: anodal transcranial direct current stimulation; Acu-TENS: acupoints-based transcutaneous electrical nerve stimulation; ARAT: Action Research Arm Test; b-tDCS: bilateral transcranial direct current stimulation; CCR: co-contraction ratio; CCFES: contralaterally controlled functional electrical stimulation; cFMA: combined Fugl-Meyer Assessment-Upper Extremity (modified to exclude the coordination, speed, and reflexes scores); CG: control group; CIMT: Constraint-Induced Movement Therapy; CN: condition; CR: conventional rehabilitation; CRO: cross-over; d: day(s); EEG: electroencephalogram; EG: experimental group; EMG: electromyography; F: female; FES: functional electrical stimulation; FMST: finger motor sequence task; FMA-UE: Fugl-Meyer Assessment-Upper Extremity; FMA-UE&LE: Fugl-Meyer Assessment-Upper Extremity and Lower Extremity; FMA-WH: Fugl-Meyer Assessment (wrist and hand parts); FTHUE-HK: Functional Test for the Hemiplegic Upper Extremity; HM: hemorrhagic stroke; Htap: Hand tapping test; IQR: interquartile range; IS: ischemic stroke; JTT: Jebsen-Taylor Hand Function Test; L: left; M: male; MAL-AOU: motor activity log – amount of use scale; MAL-QOM: motor activity log – quality of movement; MAS: Modified Ashworth Scale; MBI: Modified Barthel Index; MCT: movement cycle time; MPM: motor performance measures; MQM: movement quality measures; MS: movement smoothness; MT: motor training; m: month(s); NIHSS: National Institutes of Health Stroke Scale; NMES: neuromuscular electrical stimulation; NR: not reported; P: parallel; PNS: peripheral nerve stimulation; PV: peak velocity; R: right; ROM: range of movement; RPNS: repetitive peripheral nerve stimulation; RPSS: repetitive peripheral nerve sensory stimulation; RRV: reaching and returning velocity; SD: standard deviation; tDCS: transcranial direct current stimulation; TFI: trunk forward inclination; WMFT: Wolf Motor Function Test; y: year(s); 9HPT: 9 Hole Peg Test.

[a] Initial sample size before attrition, data after attrition were not reported and analyzed according to the intention-to-treat principle.
