# Supplementary material for: Effects of Transcranial Direct Current Stimulation Combined with Peripheral Electrical Stimulation on Upper Limb Function Recovery in Stroke: A Systematic Review and Meta-Analysis
Source: Brain Sci. 2026 Jul 27;16(8):791. doi: 10.3390/brainsci16080791 (PMC13511701; doi:10.3390/brainsci16080791)
Supplement: Supplementary file 1 [file brainsci-16-00791-s001.zip › Table S2.pdf]

Supplementary Material Table S2 (search strategy)

Details of search strategy

PubMed (n = 106)

ID	Search strings	Results
#1	"Transcranial Direct Current Stimulation"[Mesh] OR "Transcranial Direct Current Stimulation*"[tiab] OR “tDCS”[tiab] OR “non-invasive brain stimulation*”[tiab] OR “noninvasive brain stimulation*”[tiab] OR “NIBS*”[tiab] OR “anodal tDCS”[tiab] OR “cathodal tDCS”[tiab] OR “dual tDCS”[tiab] OR “bihemispheric tDCS”[tiab] OR “pair* associate* stimulation”[tiab] OR “PAS”[tiab]	46255
#2	"Upper Extremity"[Mesh] OR "Arm"[Mesh] OR "Axilla"[Mesh] OR "Elbow"[Mesh] OR "Forearm"[Mesh] OR "Hand"[Mesh] OR "Shoulder"[Mesh] OR "Wrist"[Mesh] OR "Activities of Daily Living"[Mesh] OR "Upper Extremity*"[tiab] OR “motor activity*”[tiab] OR “motor skill*”[tiab] OR “motor function*”[tiab] OR “motor recovery*”[tiab] OR “upper limb*”[tiab] OR “Arm*"[tiab] OR “Arm”[tiab] OR "Axilla*"[tiab] OR "Elbow*"[tiab] OR "Forearm*"[tiab] OR "Hand*"[tiab] OR "Shoulder*"[tiab] OR "Wrist*"[tiab] OR “dexterity*”[tiab] OR “reach*”[tiab] OR “grasp*”[tiab] OR "Activities* of Daily Living"[tiab] OR “ADL”[tiab] OR “Fugl-Meyer*”[tiab] OR “FMA”[tiab] OR “action research arm test*”[tiab] OR “ARAT*”[tiab] OR “Jebsen Taylor hand* function* test*”[tiab] OR “JTT”[tiab] OR “JTT*”[tiab] OR “wolf motor function* test*”[tiab] OR “WMFT*”[tiab]	2207141
#3	"Stroke"[Mesh] OR "Stroke, Lacunar"[Mesh] OR "Hemorrhagic Stroke"[Mesh] OR "Embolic Stroke"[Mesh] OR "Thrombotic Stroke"[Mesh] OR "Ischemic Stroke"[Mesh] OR "Stroke Rehabilitation"[Mesh] OR "Cerebrovascular Disorders"[Mesh] OR "Brain Infarction"[Mesh] OR “paresis”[Mesh] OR “hemiparesia*”[tiab] OR “hemiparesis*”[tiab] OR “hemipareses”[tiab] OR "Stroke*"[tiab] OR "Cerebrovascular Disorder*"[tiab] OR “cerebrovascular accident*”[tiab] OR “CVA”[tiab] OR “post stroke*”[tiab] OR “post-stroke*”[tiab] OR “hemiplegia*”[tiab] OR “hemiparesis*”[tiab] OR “cerebral infarct*”[tiab] OR “brain infarct*”[tiab] OR “cerebral ischemia*”[tiab] OR “brain ischemia*”[tiab] OR “brain hemorrhage*”[tiab] OR “intracranial hemorrhage*”[tiab] OR “paralysis*”[tiab] OR “palsy*”[tiab]	810122
#4	"Electric Stimulation"[Mesh] OR "Electric Stimulation Therapy"[Mesh] OR "Electric* Stimulation"[tiab] OR "Electric* Stimulation Therapy*"[tiab] OR “Electrical stimulation”[tiab] OR “electrical stimulation therapy”[tiab] OR “peripheral electric* stimulation*”[tiab] OR “peripheral electrical stimulation”[tiab] OR “PES”[tiab] OR “PES*”[tiab] OR “peripheral nerve stimulation*”[tiab] OR “peripheral neural stimulation*”[tiab] OR “PNS”[tiab] OR “PNS*”[tiab] OR “nerve	3194

	stimulation*”[tiab] OR “neural stimulation*”[tiab] OR “somatosensory stimulation*”[tiab] OR “repetitive peripheral nerve sensory stimulation*”[tiab] OR “RPSS*”[tiab] OR “electrotherapy*”[tiab]	
#5	"Transcutaneous Electric Nerve Stimulation"[Mesh] OR "Transcutaneous Electric Nerve Stimulation*"[tiab] OR “Transcutaneous Electrical Nerve Stimulation*”[tiab] OR “TENS”[tiab] OR “TENS*”[tiab] OR “cutaneous electric stimulation*”[tiab] OR “cutaneous electrical stimulation*”[tiab]	289861
#6	"Acupuncture Points"[Mesh] OR "Acupuncture Point*"[tiab] OR “acupoint*”[tiab] OR “Transcutaneous Electric* acupoint* Stimulation*"[tiab] OR “TEAS”[tiab] OR “TEAS*”[tiab] OR “Acu-TENS”[tiab] OR “Acu-TENS*”[tiab] OR “Acu TENS”[tiab] OR “Acupoint* based TENS”[tiab] OR “Acupoint*-based TENS”[tiab] OR “Acupoint TENS”[tiab] OR “Acupoints TENS”[tiab]	23680
#7	“Functional electric* stimulation*”[tiab] OR “function* electric* stimulation*”[tiab] OR “FES”[tiab] OR “FES*”[tiab] OR “functional electrical stimulation”[tiab]	14032
#8	"Electroacupuncture"[Mesh] OR "Acupuncture Therapy"[Mesh] OR “Acupuncture”[Mesh] OR "Electroacupuncture*"[tiab] OR “electric* acupuncture*”[tiab] OR “electro acupuncture”[tiab] OR “electro-acupuncture”[tiab] OR "Acupuncture Therapy*"[tiab] OR “acupuncture*”[tiab] OR “EA”[tiab] OR “EA*”[tiab]	73607
#9	“Neuromuscular electric stimulation*”[tiab] OR “Neuromuscular electrical stimulation*”[tiab] OR “NMES”[tiab] OR “NMES*”[tiab]	2888
#10	#1 AND #2 AND #3 AND #4	5
#11	#1 AND #2 AND #3 AND #5	29
#12	#1 AND #2 AND #3 AND #6	2
#13	#1 AND #2 AND #3 AND #7	32
#14	#1 AND #2 AND #3 AND #8	16
#15	#1 AND #2 AND #3 AND #9	22
	Total records identified =	106

CINAHL Complete (n =189)

ID	Search strings	Results
#1	MH "Transcranial Direct Current Stimulation" OR "Transcranial Direct Current Stimulation*" OR “tDCS” OR “non-invasive brain stimulation*” OR “noninvasive brain stimulation*” OR “NIBS*” OR “anodal tDCS” OR “cathodal tDCS” OR “dual tDCS” OR “bihemispheric tDCS” OR “pair* associate* stimulation” OR “PAS”	15140
#2	MH "Upper Extremity" OR MH "Arm" OR MH "Axilla" OR MH "Elbow" OR MH "Forearm" OR MH "Hand" OR MH "Shoulder" OR MH "Wrist" OR MH "Activities of Daily Living" OR "Upper Extremity*" OR “motor activity*” OR “motor skill*” OR “motor function*” OR “motor recovery*” OR “upper limb*” OR “Arm*" OR	559237

	“Arm” OR "Axilla*" OR "Elbow*" OR "Forearm*" OR "Hand*" OR "Shoulder*" OR "Wrist*" OR “dexterity*” OR “reach*” OR “grasp*” OR "Activities* of Daily Living" OR “ADL” OR “Fugl-Meyer*” OR “FMA” OR “action research arm test*” OR “ARAT*” OR “Jebsen Taylor hand* function* test*” OR “JTT” OR “JTT*” OR “wolf motor function* test*” OR “WMFT*”	
#3	MH "Stroke" OR MH "Stroke, Lacunar" OR MH "Hemorrhagic Stroke" OR MH "Embolic Stroke" OR MH "Thrombotic Stroke" OR MH "Ischemic Stroke" OR MH "Stroke Rehabilitation" OR MH "Cerebrovascular Disorders" OR MH "Brain Infarction" OR MH “paresis” OR “hemiparesia*” OR “hemiparesis*” OR “hemipareses” OR "Stroke*" OR "Cerebrovascular Disorder*" OR “cerebrovascular accident*” OR “CVA” OR “post stroke*” OR “post-stroke*” OR “hemiplegia*” OR “hemiparesis*” OR “cerebral infarct*” OR “brain infarct*” OR “cerebral ischemia*” OR “brain ischemia*” OR “brain hemorrhage*” OR “intracranial hemorrhage*” OR “paralysis*” OR “palsy*”	216138
#4	MH "Electric Stimulation" OR MH "Electric Stimulation Therapy" OR "Electric* Stimulation" OR "Electric* Stimulation Therapy*" OR “Electrical stimulation” OR “electrical stimulation therapy” OR “peripheral electric* stimulation*” OR “peripheral electrical stimulation” OR “PES” OR “PES*” OR “peripheral nerve stimulation*” OR “peripheral neural stimulation*” OR “PNS” OR “PNS*” OR “nerve stimulation*” OR “neural stimulation*” OR “somatosensory stimulation*” OR “repetitive peripheral nerve sensory stimulation*” OR “RPSS*” OR “electrotherapy*”	72469
#5	MH "Transcutaneous Electric Nerve Stimulation" OR "Transcutaneous Electric Nerve Stimulation*" OR “Transcutaneous Electrical Nerve Stimulation*” OR “TENS” OR “TENS*” OR “cutaneous electric stimulation*” OR “cutaneous electrical stimulation*”	42631
#6	MH "Acupuncture Points" OR "Acupuncture Point*" OR “acupoint*” OR “Transcutaneous Electric* acupoint* Stimulation*" OR “TEAS” OR “TEAS*” OR “Acu-TENS” OR “Acu-TENS*” OR “Acu TENS” OR “Acupoint* based TENS” OR “Acupoint*-based TENS” OR “Acupoint TENS” OR “Acupoints TENS”	10342
#7	MH "Electrical Stimulation, Functional" OR “Functional electric* stimulation*” OR “function* electric* stimulation*” OR “FES” OR “FES*” OR “functional electrical stimulation”	14455
#8	MH "Electroacupuncture" OR MH "Acupuncture Therapy" OR MH “Acupuncture” OR "Electroacupuncture*" OR “electric* acupuncture*” OR “electro acupuncture” OR “electro-acupuncture” OR "Acupuncture Therapy*" OR “acupuncture*” OR “EA”	40731
#9	MH "Electrical Stimulation, Neuromuscular" OR “Neuromuscular electric stimulation*” OR “Neuromuscular electrical stimulation*” OR “NMES” OR “NMES*”	7748
#10	#1 AND #2 AND #3 AND #4	84

#11	#1 AND #2 AND #3 AND #5	10
#12	#1 AND #2 AND #3 AND #6	3
#13	#1 AND #2 AND #3 AND #7	41
#14	#1 AND #2 AND #3 AND #8	7
#15	#1 AND #2 AND #3 AND #9	44
	Total records identified =	189

Embase (n = 1022)

ID	Search strings	Results
#1	'transcranial direct current stimulation'/exp OR 'tDCS (transcranial direct current stimulation)' OR 'transcranial direct current stimulation' OR 'non invasive brain stimulation'/exp OR 'noninvasive brain stimulation'/exp OR 'anodal transcranial direct current stimulation'/exp OR 'cathodal transcranial direct current stimulation'/exp OR "dual tDCS” OR “bihemispheric tDCS”	17912
#2	'upper limb'/exp OR 'upper extremity' OR 'upper limb' OR 'arm'/exp OR 'elbow'/exp OR 'forearm'/exp OR 'hand'/exp OR 'upper arm'/exp OR 'wrist'/exp OR 'shoulder'/exp OR 'arm function'/exp OR 'function, arm' OR 'upper limb function' OR 'arm function' OR 'daily life activity'/syn OR 'Fugl-Meyer assessment'/syn OR 'jebsen taylor hand function test'/exp OR 'wolf motor function test'/exp	640816
#3	'cerebrovascular accident'/syn OR 'cerebrovascular accident'/exp OR 'lacunar stroke'/syn OR 'brain hemorrhage'/syn OR 'cardioembolic stroke'/syn OR 'ischemic stroke'/syn OR 'stroke rehabilitation'/syn OR 'brain infarction'/syn OR 'paresis'/syn OR 'hemiparesis'/syn	1087419
#4	'electrostimulation'/syn OR 'electrotherapy'/syn OR 'nerve stimulation'/syn OR 'electroacupuncture'/syn OR 'functional electrical stimulation'/syn OR 'nerve cell stimulation'/syn OR 'neuromuscular electrical stimulation'/syn OR 'transcranial electrical stimulation'/syn OR 'transcutaneous electrical nerve stimulation'/syn OR 'peripheral electrical stimulation'/syn OR 'somatosensory stimulation'/exp	508522
#5	'transcutaneous electrical nerve stimulation'/syn	13891
#6	'transcutaneous electric acupoint stimulation'/syn OR 'acupuncture point'/syn OR 'teas' OR 'acu-tens' OR 'acu tens' OR 'acupoint based tens' OR 'acupoint-based tens' OR 'acupoint tens' OR 'acupoints tens'	22472
#7	'functional electrical stimulation'/syn	6107
#8	'electroacupuncture'/syn	14055
#9	'neuromuscular electrical stimulation'/syn	4501
#10	#1 AND #2 AND #3 AND #4	915
#11	#1 AND #2 AND #3 AND #5	13
#12	#1 AND #2 AND #3 AND #6	4
#13	#1 AND #2 AND #3 AND #7	51

#14	#1 AND #2 AND #3 AND #8	10
#15	#1 AND #2 AND #3 AND #9	29
	Total records identified =	1022

Cochrane Library (n = 801)

ID	Search strings	Results
#1	MeSH descriptor: [Transcranial Direct Current Stimulation] explode all trees	2384
#2	("transcranial direct-current stimulation"):ti,ab,kw OR (tDCS):ti,ab,kw OR ("transcranial electrical stimulation"):ti,ab,kw	8722
#3	(non-invasive brain stimulation):ti,ab,kw OR (noninvasive brain stimulation):ti,ab,kw OR (NIBS):ti,ab,kw	3454
#4	#1 OR #2 OR #3	10589
#5	MeSH descriptor: [Upper Extremity] explode all trees	10983
#6	MeSH descriptor: [Arm] explode all trees	1850
#7	MeSH descriptor: [Axilla] explode all trees	934
#8	MeSH descriptor: [Elbow] explode all trees	329
#9	MeSH descriptor: [Forearm] explode all trees	1828
#10	MeSH descriptor: [Hand] explode all trees	3336
#11	MeSH descriptor: [Shoulder] explode all trees	1029
#12	MeSH descriptor: [Wrist] explode all trees	529
#13	MeSH descriptor: [Activities of Daily Living] explode all trees	13767
#14	("Jebsen-Taylor Hand Function Test"):ti,ab,kw OR ("Jebsen-Taylor Hand Function Test"):ti,ab,kw OR (wolf-motor function):ti,ab,kw OR ("Fugl-Meyer"):ti,ab,kw	
#15	(upper limb):ti,ab,kw OR (upper extremity):ti,ab,kw OR (arm):ti,ab,kw OR (hand):ti,ab,kw	
#16	#5 OR #6 OR #7 OR #8 OR #9 OR #10 OR #11 OR #12 OR #13 OR #14 OR #15	263878
#17	MeSH descriptor: [Stroke] explode all trees	18937
#18	(stroke):ti,ab,kw OR (cerebrovascular accident):ti,ab,kw OR (CVA):ti,ab,kw	84689
#19	MeSH descriptor: [Paresis] explode all trees	1161
#20	(paresis):ti,ab,kw OR (hemiparesia):ti,ab,kw OR (hemiparesis):ti,ab,kw OR (hemipasese):ti,ab,kw (Word variations have been searched)	4060
#21	#17 OR #18 OR #19 OR #20	86884
#22	MeSH descriptor: [Electric Stimulation] explode all trees	2622
#23	MeSH descriptor: [Electric Stimulation Therapy] explode all trees	12074
#24	(Electric Stimulation):ti,ab,kw OR (electrical stimulation):ti,ab,kw OR (Electric Stimulation Therapy):ti,ab,kw OR (electrical stimulation therapy):ti,ab,kw (Word variations have been searched)	19574

#25	("peripheral nerve stimulation"):ti,ab,kw OR (somatosensory stimulation):ti,ab,kw OR (electrotherapy):ti,ab,kw	3258
#26	#22 OR #23 OR #24 OR #25	28682
#27	MeSH descriptor: [Transcutaneous Electric Nerve Stimulation] explode	3124
#28	("transcutaneous electric nerve stimulation"):ti,ab,kw OR ("transcutaneous electrical nerve stimulation"):ti,ab,kw OR (TENS):ti,ab,kw	42829
#29	#27 OR #28	43986
#30	MeSH descriptor: [Acupuncture Points] explode all trees	2945
#31	(acupuncture point):ti,ab,kw OR (Transcutaneous Electric acupoint Stimulation):ti,ab,kw OR (TEAS):ti,ab,kw OR (Acu TENS):ti,ab,kw OR ("acupoint"):ti,ab,kw	17878
#32	#30 OR #31	17878
#33	MeSH descriptor: [Electric Stimulation] explode all trees	2622
#34	MeSH descriptor: [Electric Stimulation Therapy] explode all trees	12074
#35	(Electric stimulation):ti,ab,kw OR (electric stimulation therapy):ti,ab,kw OR (electrical stimulation):ti,ab,kw OR (electrical stimulation therapy):ti,ab,kw	19574
#36	(functional electric stimulation):ti,ab,kw OR (functional electrical stimulation):ti,ab,kw OR (FES):ti,ab,kw	8330
#37	#33 OR #34 OR #35 OR #36	27484
#38	MeSH descriptor: [Electroacupuncture] explode all trees	1247
#39	MeSH descriptor: [Acupuncture Therapy] explode all trees	7433
#40	MeSH descriptor: [Acupuncture] explode all trees	216
#41	(electroacupuncture):ti,ab,kw OR (acupuncture therapy):ti,ab,kw OR (acupuncture treatment):ti,ab,kw OR (electric acupuncture):ti,ab,kw OR (acupuncture):ti,ab,kw (Word variations have been searched)	24014
#42	#38 OR #39 OR #40 OR #41	24213
#43	("neuromuscular electrical stimulation"):ti,ab,kw OR (neuromuscular electric stimulation):ti,ab,kw OR (NMES):ti,ab,kw	2704
#44	#4 AND #16 AND #21 AND #26	358
#45	#4 AND #16 AND #21 AND #29	58
#46	#4 AND #16 AND #21 AND #32	8
#47	#4 AND #16 AND #21 AND #37	345
#48	#4 AND #16 AND #21 AND #42	12
#49	#4 AND #16 AND #21 AND #43	20
	Total records identified =	801

Scopus (n = 645)

ID	Search strings	Results

#1	TITLE-ABS-KEY ("Transcranial Direct Current Stimulation" OR "Transcranial Direct Current Stimulation*" OR "tDCS" OR "non-invasive brain stimulation*" OR "noninvasive brain stimulation*" OR "NIBS*" OR "anodal tDCS" OR "cathodal tDCS" OR "dual tDCS" OR "bihemispheric tDCS" OR "pair* associate* stimulation" OR "PAS") AND TITLE-ABS-KEY ("Upper Extremity" OR "Arm" OR "Axilla" OR "Elbow" OR "Forearm" OR "Hand" OR "Shoulder" OR "Wrist" OR "Activities of Daily Living" OR "Upper Extremity*" OR "motor activity*" OR "motor skill*" OR "motor function*" OR "motor recovery*" OR "upper limb*" OR "Arm*" OR "Arm" OR "Axilla*" OR "Elbow*" OR "Forearm*" OR "Hand*" OR "Shoulder*" OR "Wrist*" OR "dexterity*" OR "reach*" OR "grasp*" OR "Activities* of Daily Living" OR "ADL" OR "Fugl-Meyer*" OR "FMA" OR "action research arm test*" OR "ARAT*" OR "Jebsen Taylor hand* function* test*" OR "JTT" OR "JTT*" OR "wolf motor function* test*" OR "WMFT*") AND TITLE-ABS-KEY ("Stroke" OR "Stroke, Lacunar" OR "Hemorrhagic Stroke" OR "Embolic Stroke" OR "Thrombotic Stroke" OR "Ischemic Stroke" OR "Stroke Rehabilitation" OR "Cerebrovascular Disorders" OR "Brain Infarction" OR "paresis" OR "hemiparesia*" OR "hemiparesis*" OR "hemipareses" OR "Stroke*" OR "Cerebrovascular Disorder*" OR "cerebrovascular accident*" OR "CVA" OR "post stroke*" OR "post-stroke*" OR "hemiplegia*" OR "hemiparesis*" OR "cerebral infarct*" OR "brain infarct*" OR "cerebral ischemia*" OR "brain ischemia*" OR "brain hemorrhage*" OR "intracranial hemorrhage*" OR "paralysis*" OR "palsy*") AND TITLE-ABS-KEY ("Electric Stimulation" OR "Electric Stimulation Therapy" OR "Electric* Stimulation" OR "Electric* Stimulation Therapy*" OR "Electrical stimulation" OR "electrical stimulation therapy" OR "peripheral electric* stimulation*" OR "peripheral electrical stimulation" OR "PES" OR "PES*" OR "peripheral nerve stimulation*" OR "peripheral neural stimulation*" OR "PNS" OR "PNS*" OR "nerve stimulation*" OR "neural stimulation*" OR "somatosensory stimulation*" OR "repetitive peripheral nerve sensory stimulation*" OR "RPSS*" OR "electrotherapy*")	388
#2	TITLE-ABS-KEY ("Transcranial Direct Current Stimulation" OR "Transcranial Direct Current Stimulation*" OR "tDCS" OR "non-invasive brain stimulation*" OR "noninvasive brain stimulation*" OR "NIBS*" OR "anodal tDCS" OR "cathodal tDCS" OR "dual tDCS" OR "bihemispheric tDCS" OR "pair* associate* stimulation" OR "PAS") AND TITLE-ABS-KEY ("Upper Extremity" OR "Arm" OR "Axilla" OR "Elbow" OR "Forearm" OR "Hand" OR "Shoulder" OR "Wrist" OR "Activities of Daily Living" OR "Upper Extremity*" OR "motor activity*" OR "motor skill*" OR "motor function*" OR "motor recovery*" OR "upper limb*" OR "Arm*" OR "Arm" OR "Axilla*" OR "Elbow*" OR "Forearm*" OR "Hand*" OR "Shoulder*" OR "Wrist*" OR "dexterity*" OR "reach*" OR "grasp*" OR "Activities* of Daily Living" OR "ADL" OR "Fugl-Meyer*"	86

	OR "FMA" OR "action research arm test*" OR "ARAT*" OR "Jebsen Taylor hand* function* test*" OR "JTT" OR "JTT*" OR "wolf motor function* test*" OR "WMFT*") AND TITLE-ABS-KEY ("Stroke" OR "Stroke, Lacunar" OR "Hemorrhagic Stroke" OR "Embolic Stroke" OR "Thrombotic Stroke" OR "Ischemic Stroke" OR "Stroke Rehabilitation" OR "Cerebrovascular Disorders" OR "Brain Infarction" OR "paresis" OR "hemiparesia*" OR "hemiparesis*" OR "hemipareses" OR "Stroke*" OR "Cerebrovascular Disorder*" OR "cerebrovascular accident*" OR "CVA" OR "post stroke*" OR "post-stroke*" OR "hemiplegia*" OR "hemiparesis*" OR "cerebral infarct*" OR "brain infarct*" OR "cerebral ischemia*" OR "brain ischemia*" OR "brain hemorrhage*" OR "intracranial hemorrhage*" OR "paralysis*" OR "palsy*") AND TITLE-ABS-KEY ("Transcutaneous Electric Nerve Stimulation" OR "Transcutaneous Electric Nerve Stimulation*" OR "Transcutaneous Electrical Nerve Stimulation*" OR "TENS" OR "TENS*" OR "cutaneous electric stimulation*" OR "cutaneous electrical stimulation*")	
#3	TITLE-ABS-KEY ("Transcranial Direct Current Stimulation" OR "Transcranial Direct Current Stimulation*" OR "tDCS" OR "non-invasive brain stimulation*" OR "noninvasive brain stimulation*" OR "NIBS*" OR "anodal tDCS" OR "cathodal tDCS" OR "dual tDCS" OR "bihemispheric tDCS" OR "pair* associate* stimulation" OR "PAS") AND TITLE-ABS-KEY ("Upper Extremity" OR "Arm" OR "Axilla" OR "Elbow" OR "Forearm" OR "Hand" OR "Shoulder" OR "Wrist" OR "Activities of Daily Living" OR "Upper Extremity*" OR "motor activity*" OR "motor skill*" OR "motor function*" OR "motor recovery*" OR "upper limb*" OR "Arm*" OR "Arm" OR "Axilla*" OR "Elbow*" OR "Forearm*" OR "Hand*" OR "Shoulder*" OR "Wrist*" OR "dexterity*" OR "reach*" OR "grasp*" OR "Activities* of Daily Living" OR "ADL" OR "Fugl-Meyer*" OR "FMA" OR "action research arm test*" OR "ARAT*" OR "Jebsen Taylor hand* function* test*" OR "JTT" OR "JTT*" OR "wolf motor function* test*" OR "WMFT*") AND TITLE-ABS-KEY ("Stroke" OR "Stroke, Lacunar" OR "Hemorrhagic Stroke" OR "Embolic Stroke" OR "Thrombotic Stroke" OR "Ischemic Stroke" OR "Stroke Rehabilitation" OR "Cerebrovascular Disorders" OR "Brain Infarction" OR "paresis" OR "hemiparesia*" OR "hemiparesis*" OR "hemipareses" OR "Stroke*" OR "Cerebrovascular Disorder*" OR "cerebrovascular accident*" OR "CVA" OR "post stroke*" OR "post-stroke*" OR "hemiplegia*" OR "hemiparesis*" OR "cerebral infarct*" OR "brain infarct*" OR "cerebral ischemia*" OR "brain ischemia*" OR "brain hemorrhage*" OR "intracranial hemorrhage*" OR "paralysis*" OR "palsy*") AND TITLE-ABS-KEY ("Acupuncture Points" OR "Acupuncture Point*" OR "acupoint*" OR "Transcutaneous Electric* acupoint* Stimulation*" OR "TEAS" OR "TEAS*" OR "Acu-TENS" OR "Acu-TENS*" OR "Acu	8

	TENS" OR "Acupoint* based TENS" OR "Acupoint*-based TENS" OR "Acupoint TENS" OR "Acupoints TENS")	
#4	TITLE-ABS-KEY ("Transcranial Direct Current Stimulation" OR "Transcranial Direct Current Stimulation*" OR "tDCS" OR "non-invasive brain stimulation*" OR "noninvasive brain stimulation*" OR "NIBS*" OR "anodal tDCS" OR "cathodal tDCS" OR "dual tDCS" OR "bihemispheric tDCS" OR "pair* associate* stimulation" OR "PAS") AND TITLE-ABS-KEY ("Upper Extremity" OR "Arm" OR "Axilla" OR "Elbow" OR "Forearm" OR "Hand" OR "Shoulder" OR "Wrist" OR "Activities of Daily Living" OR "Upper Extremity*" OR "motor activity*" OR "motor skill*" OR "motor function*" OR "motor recovery*" OR "upper limb*" OR "Arm*" OR "Arm" OR "Axilla*" OR "Elbow*" OR "Forearm*" OR "Hand*" OR "Shoulder*" OR "Wrist*" OR "dexterity*" OR "reach*" OR "grasp*" OR "Activities* of Daily Living" OR "ADL" OR "Fugl-Meyer*" OR "FMA" OR "action research arm test*" OR "ARAT*" OR "Jebsen Taylor hand* function* test*" OR "JTT" OR "JTT*" OR "wolf motor function* test*" OR "WMFT*") AND TITLE-ABS-KEY ("Stroke" OR "Stroke, Lacunar" OR "Hemorrhagic Stroke" OR "Embolic Stroke" OR "Thrombotic Stroke" OR "Ischemic Stroke" OR "Stroke Rehabilitation" OR "Cerebrovascular Disorders" OR "Brain Infarction" OR "paresis" OR "hemiparesia*" OR "hemiparesis*" OR "hemipareses" OR "Stroke*" OR "Cerebrovascular Disorder*" OR "cerebrovascular accident*" OR "CVA" OR "post stroke*" OR "post-stroke*" OR "hemiplegia*" OR "hemiparesis*" OR "cerebral infarct*" OR "brain infarct*" OR "cerebral ischemia*" OR "brain ischemia*" OR "brain hemorrhage*" OR "intracranial hemorrhage*" OR "paralysis*" OR "palsy*") AND TITLE-ABS-KEY ("Functional electric* stimulation*" OR "function* electric* stimulation*" OR "FES" OR "FES*" OR "functional electrical stimulation")	81
#5	TITLE-ABS-KEY ("Transcranial Direct Current Stimulation" OR "Transcranial Direct Current Stimulation*" OR "tDCS" OR "non-invasive brain stimulation*" OR "noninvasive brain stimulation*" OR "NIBS*" OR "anodal tDCS" OR "cathodal tDCS" OR "dual tDCS" OR "bihemispheric tDCS" OR "pair* associate* stimulation" OR "PAS") AND TITLE-ABS-KEY ("Upper Extremity" OR "Arm" OR "Axilla" OR "Elbow" OR "Forearm" OR "Hand" OR "Shoulder" OR "Wrist" OR "Activities of Daily Living" OR "Upper Extremity*" OR "motor activity*" OR "motor skill*" OR "motor function*" OR "motor recovery*" OR "upper limb*" OR "Arm*" OR "Arm" OR "Axilla*" OR "Elbow*" OR "Forearm*" OR "Hand*" OR "Shoulder*" OR "Wrist*" OR "dexterity*" OR "reach*" OR "grasp*" OR "Activities* of Daily Living" OR "ADL" OR "Fugl-Meyer*" OR "FMA" OR "action research arm test*" OR "ARAT*" OR "Jebsen Taylor hand* function* test*" OR "JTT" OR "JTT*" OR "wolf motor	41

	function* test*" OR "WMFT*") AND TITLE-ABS-KEY ("Stroke" OR "Stroke, Lacunar" OR "Hemorrhagic Stroke" OR "Embolic Stroke" OR "Thrombotic Stroke" OR "Ischemic Stroke" OR "Stroke Rehabilitation" OR "Cerebrovascular Disorders" OR "Brain Infarction" OR "paresis" OR "hemiparesia*" OR "hemiparesis*" OR "hemipareses" OR "Stroke*" OR "Cerebrovascular Disorder*" OR "cerebrovascular accident*" OR "CVA" OR "post stroke*" OR "post-stroke*" OR "hemiplegia*" OR "hemiparesis*" OR "cerebral infarct*" OR "brain infarct*" OR "cerebral ischemia*" OR "brain ischemia*" OR "brain hemorrhage*" OR "intracranial hemorrhage*" OR "paralysis*" OR "palsy*") AND TITLE-ABS-KEY ("Electroacupuncture" OR "Acupuncture Therapy" OR "Acupuncture" OR "Electroacupuncture*" OR "electric* acupuncture*" OR "electro acupuncture" OR "electro-acupuncture" OR "Acupuncture Therapy*" OR "acupuncture*" OR "EA")	
#6	TITLE-ABS-KEY ("Transcranial Direct Current Stimulation" OR "Transcranial Direct Current Stimulation*" OR "tDCS" OR "non-invasive brain stimulation*" OR "noninvasive brain stimulation*" OR "NIBS*" OR "anodal tDCS" OR "cathodal tDCS" OR "dual tDCS" OR "bihemispheric tDCS" OR "pair* associate* stimulation" OR "PAS") AND TITLE-ABS-KEY ("Upper Extremity" OR "Arm" OR "Axilla" OR "Elbow" OR "Forearm" OR "Hand" OR "Shoulder" OR "Wrist" OR "Activities of Daily Living" OR "Upper Extremity*" OR "motor activity*" OR "motor skill*" OR "motor function*" OR "motor recovery*" OR "upper limb*" OR "Arm*" OR "Arm" OR "Axilla*" OR "Elbow*" OR "Forearm*" OR "Hand*" OR "Shoulder*" OR "Wrist*" OR "dexterity*" OR "reach*" OR "grasp*" OR "Activities* of Daily Living" OR "ADL" OR "Fugl-Meyer*" OR "FMA" OR "action research arm test*" OR "ARAT*" OR "Jebsen Taylor hand* function* test*" OR "JTT" OR "JTT*" OR "wolf motor function* test*" OR "WMFT*") AND TITLE-ABS-KEY ("Stroke" OR "Stroke, Lacunar" OR "Hemorrhagic Stroke" OR "Embolic Stroke" OR "Thrombotic Stroke" OR "Ischemic Stroke" OR "Stroke Rehabilitation" OR "Cerebrovascular Disorders" OR "Brain Infarction" OR "paresis" OR "hemiparesia*" OR "hemiparesis*" OR "hemipareses" OR "Stroke*" OR "Cerebrovascular Disorder*" OR "cerebrovascular accident*" OR "CVA" OR "post stroke*" OR "post-stroke*" OR "hemiplegia*" OR "hemiparesis*" OR "cerebral infarct*" OR "brain infarct*" OR "cerebral ischemia*" OR "brain ischemia*" OR "brain hemorrhage*" OR "intracranial hemorrhage*" OR "paralysis*" OR "palsy*") AND TITLE-ABS-KEY ("Neuromuscular electric stimulation*" OR "Neuromuscular electrical stimulation*" OR "NMES" OR "NMES*")	41
	Total records identified =	645

Web of Science (n = 375)

ID	Search strings	Results
#1	"Transcranial Direct Current Stimulation" OR "Transcranial Direct Current Stimulation*" OR "tDCS" OR "non-invasive brain stimulation*" OR "noninvasive brain stimulation*" OR "NIBS*" OR "anodal tDCS" OR "cathodal tDCS" OR "dual tDCS" OR "bihemispheric tDCS" OR "pair* associate* stimulation" OR "PAS"	65404
#2	"Upper Extremity" OR "Arm" OR "Axilla" OR "Elbow" OR "Forearm" OR "Hand" OR "Shoulder" OR "Wrist" OR "Activities of Daily Living" OR "Upper Extremity*" OR "motor activity*" OR "motor skill*" OR "motor function*" OR "motor recovery*" OR "upper limb*" OR "Arm*" OR "Arm" OR "Axilla*" OR "Elbow*" OR "Forearm*" OR "Hand*" OR "Shoulder*" OR "Wrist*" OR "dexterity*" OR "reach*" OR "grasp*" OR "Activities* of Daily Living" OR "ADL" OR "Fugl-Meyer*" OR "FMA" OR "action research arm test*" OR "ARAT*" OR "Jebsen Taylor hand* function* test*" OR "JTT" OR "JTT*" OR "wolf motor function* test*" OR "WMFT*"	4459236
#3	"Stroke" OR "Stroke, Lacunar" OR "Hemorrhagic Stroke" OR "Embolic Stroke" OR "Thrombotic Stroke" OR "Ischemic Stroke" OR "Stroke Rehabilitation" OR "Cerebrovascular Disorders" OR "Brain Infarction" OR "paresis" OR "hemiparesia*" OR "hemiparesis*" OR "hemipareses" OR "Stroke*" OR "Cerebrovascular Disorder*" OR "cerebrovascular accident*" OR "CVA" OR "post stroke*" OR "post-stroke*" OR "hemiplegia*" OR "hemiparesis*" OR "cerebral infarct*" OR "brain infarct*" OR "cerebral ischemia*" OR "brain ischemia*" OR "brain hemorrhage*" OR "intracranial hemorrhage*" OR "paralysis*" OR "palsy*"	730585
#4	"Electric Stimulation" OR "Electric Stimulation Therapy" OR "Electric* Stimulation" OR "Electric* Stimulation Therapy*" OR "Electrical stimulation" OR "electrical stimulation therapy" OR "peripheral electric* stimulation*" OR "peripheral electrical stimulation" OR "PES" OR "PES*" OR "peripheral nerve stimulation*" OR "peripheral neural stimulation*" OR "PNS" OR "PNS*" OR "nerve stimulation*" OR "neural stimulation*" OR "somatosensory stimulation*" OR "repetitive peripheral nerve sensory stimulation*" OR "RPSS*" OR "electrotherapy*"	517766
#5	"Transcutaneous Electric Nerve Stimulation" OR "Transcutaneous Electric Nerve Stimulation*" OR "Transcutaneous Electrical Nerve Stimulation*" OR "TENS" OR "TENS*" OR "cutaneous electric stimulation*" OR "cutaneous electrical stimulation*"	1128014

#6	"Acupuncture Points" OR "Acupuncture Point*" OR "acupoint*" OR "Transcutaneous Electric* acupoint* Stimulation*" OR "TEAS" OR "TEAS*" OR "Acu-TENS" OR "Acu-TENS*" OR "Acu TENS" OR "Acupoint* based TENS" OR "Acupoint*-based TENS" OR "Acupoint TENS" OR "Acupoints TENS"	24979
#7	"Functional electric* stimulation*" OR "function* electric* stimulation*" OR "FES" OR "FES*" OR "functional electrical stimulation"	154645
#8	"Electroacupuncture" OR "Acupuncture Therapy" OR "Acupuncture" OR "Electroacupuncture*" OR "electric* acupuncture*" OR "electro acupuncture" OR "electro-acupuncture" OR "Acupuncture Therapy*" OR "acupuncture*" OR "EA"	86245
#9	"Neuromuscular electric stimulation*" OR "Neuromuscular electrical stimulation*" OR "NMES" OR "NMES*"	4009
#10	#1 AND #2 AND #3 AND #4	251
#11	#1 AND #2 AND #3 AND #5	31
#12	#1 AND #2 AND #3 AND #6	3
#13	#1 AND #2 AND #3 AND #7	37
#14	#1 AND #2 AND #3 AND #8	22
#15	#1 AND #2 AND #3 AND #9	31
	Total records identified =	375
